# Climate change adaptation and the role of fuel subsidies: An empirical bio-economic modeling study for an artisanal open-access fishery

**DOI:** 10.1371/journal.pone.0220433

**Published:** 2019-08-21

**Authors:** Kira Lancker, Anna-Lena Deppenmeier, Teferi Demissie, Jörn O. Schmidt

**Affiliations:** 1 Biodiversity Economics, German Center for Integrative Biodiversity Halle-Jena-Leipzig (iDiv), Leipzig, Germany; 2 Meteorology and Air Quality Department, Wageningen University, Wageningen, The Netherlands; 3 R&D Weather and Climate Modeling, Royal Netherlands Meteorological Institute (KNMI), De Bilt, The Netherlands; 4 NORCE Climate, NORCE Norwegian Research Centre AS, Bergen, Norway; 5 CGIAR Research Program on Climate Change, Agriculture and Food Security (CCAFS) East Africa, Addis Ababa, Ethiopia; 6 Department of Economics, Christian-Albrechts University of Kiel, Kiel, Germany; Universite du Quebec a Montreal, CANADA

## Abstract

Climate change can severely impact artisanal fisheries and affect the role they play in food security. We study climate change effects on the triple bottom line of ecological productivity, fishers’ incomes, and fish consumption for an artisanal open-access fishery. We develop and apply an empirical, stochastic bio-economic model for the Senegalese artisanal purse seine fishery on small pelagic fish and compare the simulated fishery’s development using four climate projections and two policy scenarios. We find that economic processes of adaptation may amplify the effects of climate variations. The regions’ catch potential increases with climate change, induced by stock distribution changes. However, this outcome escalates over-fishing, whose effects outpace the incipiently favorable climate change effects under three of the four climate projections. Without policy action, the fishery is estimated to collapse in 2030–2035 on average over 1000 runs. We propose an easily implementable and overall welfare-increasing intervention: reduction of fuel subsidies. If fuel subsidies were abolished, ecological sustainability as well as the fishery’s welfare contribution would increase regardless of the climate projection.

## 1 Introduction

Fish constitutes one of the most important natural resources with respect to food security, protein intake, and income generation. More than 20 kg of fish is consumed per capita per year, and in 2018, more than 40 million people earned their livelihoods in capture fisheries [1, 2]. The artisanal sector accounts for around 90% of this employment. Consumers are highly dependent on fish as a protein source [3, 4]. Artisanal fishing communities are particularly vulnerable to climate change [5] and economic impacts may be severe [6–8]. Upwelling-favorable winds are expected to increase in the north, decrease in the south [9, 10], and thus, change primary productivity. Cheung et al. [7] reported an increase in global mean water temperature at time of catch of 0.19°C per decade, due to fish stock redistribution. Artisanal fisheries suffer from a relatively small fishing range around their landing port, such that a redistribution of their target stock may impact their fishing success. For 14 northwest African countries, Lam et al. [11] projected an average climate change-induced decrease in landed fish of 25.9% between 2000 and 2050, associated with the loss of 50% of available jobs in the region’s fisheries. Other countries could benefit from enhanced catch potential [6, 12].

Owing to the complexity and global nature of the research, studies rely on simplifying assumptions, such as constant per capita consumption and landings according to biological catch potential or economic growth [6, 7, 11, 13]. However, changes may be dampened or intensified when fishers and consumers adapt. In open-access artisanal fisheries, in which local market feedbacks are typically strong, such adaptation processes are important. For example, fishers may adapt by changing the used amounts of fuel, capital, and labor in fishing. As a result, over-fishing may increase or decrease with rising harvest potential. Non-linear changes in equilibrium harvest and fish prices arise. While this is scarcely analyzed in fisheries models [14], Osborne et al. [15] explicitly modeled adaptation in a crop model, which turned negative impacts of climate change into positive crop yield gains. Apart from climate change, economic changes over time also shape future fish harvest and prices. Market development and urbanization change consumer dependence on fish [16]. Fishing costs depend on the development of fuel (and thus, oil) prices, which alters the amount fished and the distance traveled by fishing boats [17, 18]. The importance of fuel for fishing has been underlined in the literature [18–20]. Fuel costs constitute one-third of global output value [18]. On the one hand, higher fuel prices, all else equal, decrease harvest. On the other hand, technological progress likely increases harvest efficiency [21].

Both climate projections and economic changes are important for policymakers to consider. Therefore, this study aims to simulate the impact of climatic changes on an artisanal open-access fishery under endogenous economic adaptation and (exogenous) economic development. High uncertainty exists with respect to climate change projections, particularly those concerning the amplitude of climate change and the extent of climate variability. Hence, for a robust understanding of climate change adaptation, it is necessary to compare how a fishery develops under different projections and to explore which climate scenario traits are important determinants.

We develop an empirical bio-economic model for the *Sardinella aurita* fishery by artisanal purse seines in Senegal, which takes into account climate effects on reproduction of the fish stock, productivity of harvesting and feedbacks on local input and output markets. Climate has a dynamic effect on yearly stock growth through changes in upwelling intensity, but also an effect on catchability, and thus, fishing pressure through fish stock distribution. We simulate the development of biomass, harvest, and prices and we compare outcomes concerning the fishery’s contribution to food and income security. To explore mitigation options, we suggest an easily implementable and overall welfare-increasing intervention: reduction of fuel subsidies. The price for fossil fuels shapes the adaptation process in fisheries. Fuel subsidies constitute one of the most widely used policy instruments: Sumaila et al. [22] estimate that an annual 7.7 billion US Dollars of fuel subsidies are paid to fisheries globally. It has been argued that fisheries that are relevant for food security may need fuel subsidies to help maintain high effort, high catches and low fish prices for consumers [23, 24]. By contrast, several authors have found a detrimental dynamic impact on fish stocks [25, 26]. Fishing fleets benefit from a subsidy decrease if the stock effect is large enough [27]. Against this background, we evaluate how climate change outcomes for a fishery could be shaped if fuel subsidies were abolished. Our results are informative for policymakers, as the findings shed light on the effect of expected climate change impacts and mitigation options while considering endogenous adaptation of actors. In summary, the study aims and achieves to investigate how a fishery may be impacted by climate change when both economic adaptation and development is considered, and continues to explore counteracting policy measures.

The next section describes the data and methods used in the biological and economic model parts, as well as the simulation strategy and climate scenarios. The results are presented and discussed in Section 3.

## 2 Methods and materials

### 2.1 Biology and climate impacts on stock growth of Senegalese *Sardinella aurita*

*Sardinella aurita* is a migratory small pelagic schooling species. The species occurs along the continental shelf between Morocco and Guinea Bissau [28]. Recent findings support the separability of the stock into sub-populations [29–33], with one group along Senegal and Southern Mauritania. Spawning in Senegal peaks in fall and spring [34]. We focus our research on this subpopulation. We tested for an effect of the more Northern Mauritanian sub-population on Senegalese catches, but found no significant effect.

To estimate climate impacts on biomass growth, we use stock assessment data for *Sardinella aurita* by the Food and Agriculture organization (FAO) working group on the assessment of small pelagic fish off Northwest Africa. In the years 1995–2006, catch independent surveys were carried out in the fourth quarter of each year by the research vessel R/V Dr FRIDTJOF NANSEN [35–40]. National surveys provide estimates for 2007–2009 [41–43]. The working group used regression models to account for occasional regional gaps. We present a figure containing yearly abundance estimates in Section A of the supporting information S1 File. The development over time shows substantial variability, which has been attributed to upwelling variability and associated variations in primary productivity [29, 34, 44].

The habitat is characterized by high productivity owing to its upwelling system [45]. Alongshore wind stress brings cool, nutrient-rich water to the surface [9]. In such upwelling systems, small pelagic reproduction has been found to depend mainly on two factors: retention of larvae and primary productivity. Both are influenced by wind conditions [32, 46–51]. On the one hand, according to Thiaw et al. [34], the lagged spring and autumn upwelling indexes have a significant positive effect on *Sardinella aurita* abundance, because they spur primary productivity. On the other hand, wind stress can influence retention negatively, because larvae drift off to areas where they cannot survive [49, 52]. Owing to the broad continental shelf in the main spawning area, retention is high in general [48]. We use the ERA Interim Reanalysis product, available from the European Centre for Medium-Range Weather Forecasts [53], to calculate a seasonal coastal upwelling index (CUI) from wind stress using standard procedures [9] (see Section B of the S1 File). We average over the area between 13.5–16°N and 17.25–18°W. Our index shows the typical regional pattern: upwelling is strongest in winter, and subsides between June and October.

We estimate the effect of upwelling on yearly stock growth (surplus stock production), defined as the biomass difference between consecutive years plus catches. Data on annual Senegalese and Gambian catches for the period 1995–2013 are taken from the FAO assessment report [54]. We add reported catches by legal foreign trawlers from the “Sea around us” data-base [55, 56]. The share of the Senegalese purse seine fleet in total harvest fluctuates around 90%, because the stocks’ main habitat is the Senegalese exclusive economic zone, to which other fleets have limited access. A summary of the dataset used in estimation is provided in Section C of the S1 File.

We assume a standard logistic growth model, with spawning stock biomass (SSB) lagged by 1 year, *x*_*y*−1_, where *y* denotes the year in which biomass growth is observed. The intrinsic growth rate *r* + *f*(*UP*_*y*_) depends on upwelling intensity *UP*_*y*_, and we use *K* to denote the (constant) carrying capacity for the fish stock. Adding a normally distributed shock $\varepsilon_{y}^{s}$, net growth *g*_*y*_ of the fish stock in year *y* is given by

$$g_{y}=(r+f(UP_{y}))\;x_{y-1}\;(1-\frac{x_{y-1}}{K})+\varepsilon_{y}^{s}$$ (1)

The error term $\varepsilon_{y}^{s}$ is approximately normally distributed with an estimated standard deviation of 80.29 kt per year. The function *f*(*UP*_*y*_) denotes a linear sum of upwelling impacts, in which various structures are tested. We consider alternative specifications with respect to lag structure, seasons, SST, an alternative upwelling index based on SST, and the size of the biomass or biomass growth of the Mauritanian stock group to check for migration flows. We do not find a direct impact of temperature on recruitment, even though the literature finds a non-linear relationship, and a positive impact from SST up to 28.5°C [57]. We also test alternatives with weather impacts on the carrying capacity, but models with environmental effects on the natural growth rate generally perform better than do models with environmental effects on carrying capacity *K*, or on the quadratic term in general. The model is chosen based on the Bayesian information criterion and adjusted *R*^2^. In the chosen model, the 1-year lagged fall (October–December) and winter (January–March) CUI have a linear influence on the natural growth rate:

$$r+f\left(UP_{y}\right)=r+r_{Cw}CUI_{winter,y-1}+r_{Cf}CUI_{fall,y-1}$$ (2)

The model features an adjusted *R*^2^ of 92%, which is non-surprising considering that only 14 observations (1996–2009) are available and upwelling has been established as the main driver by previous research. The inclusion of upwelling explains 28% of the variation compared to the model without climate impacts. No issues are detected in tests for serial correlation, heteroscedasticity, and skewness at the 5% level. An augmented Dickey–Fuller test gives a test statistic of -3.874 for logged growth and a critical 1%-level value of -3.750, such that we find the data to be stationary. In the following part, we present the results and marginal effects. Section D of the S1 File includes technical details as well as alternative specifications.

**Fig 1:**
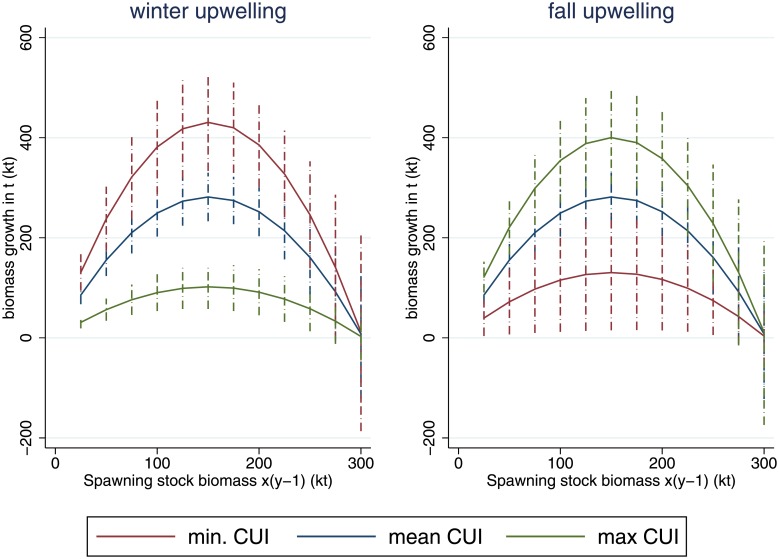
Sensitivity of biomass growth to a simultaneous variation of SSB (*x*_*y*−1_) and upwelling. Dashed lines mark the 95% confidence interval variation.

The carrying capacity is estimated as 302 kt, and is significant at the 1% level. The positive impact from fall upwelling, significant at 5%, is partly canceled out by the negative impact from winter upwelling (significant at the 1% level). The latter could be linked to lower retention and colder water temperatures unfavorable for the autumn larvae, which would delay spring spawning. Thiaw et al. [34] also reported a negative sign for winter upwelling, although it was insignificant. As the mean SSB *x*_*y*−1_ (199.5 kt) lies above the maximum sustainable yield, the marginal effect at the means of an increasing SSB on stock growth is negative at -1.21 kt. An increase in the upwelling indicators by 1 $\frac{m^{3}}{s}$ per 100 m of coastline leads to a growth reduction of 18.49 kt concerning winter upwelling, and an increase of 20.61 kt for fall upwelling. Fig 1 shows the stock growth effect of varying one environmental variable between its minimum and maximum observed levels for fall and winter upwelling respectively, where the other upwelling variable is held constant at its mean. Observed values lie between 0.2509 and 0.4103$\frac{m^{3}}{s}$ per meter of coastline for winter upwelling and 0.1243–0.2418$\frac{m^{3}}{s}$ per meter of coastline for fall upwelling. The graph shows that stock growth varies slightly more with winter upwelling than with fall upwelling. Predicted growth at maximum sustainable yield SSB ranges between 100 and 430 kt (66–285% of SSB) within the study period, based only on upwelling variation.

We use available harvest and climate data for 2010–2014 to simulate the development of the stock for 2010–2014. The model provides plausible results (see Section A of the S1 File). These predictions are used in the following economic modeling part.

### 2.2 Economy and climate impacts on harvesting costs

Fishers’ harvesting decisions are affected by climatic conditions, prices for fish, fuel and labor, technological progress, and the stock level. We use a similar economic dataset to previous studies [58, 59]. We model purse seines targeting *Sardinella aurita* in four Senegalese regions, namely, Thiès Sud, Thiès Nord, Fleuve, and the Dakar peninsula called Cap Vert, and we aggregate the results afterwards. Thus, we limit the included catches to those landed in Senegal. Part of the catch landed in Fleuve may stem from Mauritanian waters. These catches are included, as long as they are landed in Senegal, and thus can be assumed to contribute to Senegalese fishers and consumers rents. We use monthly regional data on purse seine catches (tons), prices (FCFA/kg), and effort (days at sea, DAS) for 2001–2013, provided by the Centre of Oceanographic Research of Dakar-Thiaroye (CRODT). Purse seines exhibited an annual catch share of more than 80% for 2001–2013 in all regions except Thiès Sud (57%). Price data after 2013 are not available to us, which is a limiting factor for the time period covered by our estimation dataset. All prices are given in units of 2010-level FCFA, which is the the Senegalese currency Franc de la Communauté Financière d’Afrique, after deflation with yearly World Bank Consumer Price Index data [60]. We use regional *Sardinella aurita* prices, but fill in gaps with prices from the neighboring region or alternatively the price for *Sardinella maderensis*, the closest substitute (4% of observations). We prolong the time series of annual biomass data (cf., Section 2.1) by using our own stock predictions for 2010–2013. This allows us to make use of 187 additional data points, which is desirable in particular for a more robust estimation of time trends. It is also important when looking at fuel impacts, as fuel prices typically are more variable over the long term. The estimation results for the time period 2001–2009, including only observed biomass levels, and further alternative specifications are provided in S7 Section. Monthly local fuel price data are obtained from the annual reports of the Directorate of Maritime Fisheries [61–70]. Prices are given in units of FCFA/liter. Local fuel prices and global prices for the internationally traded Brent crude oil are highly correlated. A strong upward trend can be observed until 2008, after which fuel prices fall to the level of 2005, followed again by a gradual increase. For climate impacts, we again use the ERA Interim Reanalysis product with a monthly time scale. We average over regional coordinate boxes between 13.5–16.5°N and 17.25–18.75°W. The dataset is summarized in Section C of the S1 File.

Supply and demand are simultaneously estimated via the reduced form using robust standard errors. We estimate 32 parameters in three equations from 561 observations. These are monthly regional observations during 2009–2013 with few, unsystematic gaps. A Bayesian information criterion of 4056 and an *R*^2^ of 32–34% indicate a good model fit. Sections E and F of the S1 File provide technical details on the model and estimation.

We assume an iso-elastic inverse demand function for fresh *Sardinella aurita*. Our estimates show that inverse demand is decreasing and convex in harvest (significant at the 1% level). This means that consumers are willing to pay a higher price if fish supply is scarce, and the marginal effect decreases with the quantity supplied. This is in accordance with the literature finding that fish is sold mainly in local markets [13, 71, 72]. *Sardinella aurita* is an important consumption good for these local markets, and consumers respond to quantity changes. Furthermore, the price elasticity of demand is found to depend negatively on population density, a result that is significant at the 1% level. This is a sensible finding, as population density can be taken as a proxy for market size. Larger markets, in turn, spur the supply of various products at affordable prices, and reduce consumers’ dependence on this particular fish. We assume that a linear time trend affects prices. Our empirical estimates show that prices are subject to a monthly positive linear time trend of 0.2%, an estimate that is significant at the 10% level.

The harvesting technology is modeled as a constant returns-to-scale Cobb–Douglas function from a capital–labor composite and fuel. This means that fishers can substitute between fuel, capital, and labor: If one of these inputs becomes more expensive, the fisher is able to partly replace it with other inputs. Several estimation studies [73–77], concur that substitution possibilities exist in fisheries. We choose a substitution elasticity of one to keep the model simple and tractable. Harvest production is assumed to depend positively on biomass. At larger biomass values, catches become easier, as fish are located more easily. The “catchability” parameter is used for scaling. A linear time trend on catchability reflects unobserved changes over time. Monthly dummies are introduced to represent regular harvest seasonality, for example, owing to biomass growth, northward stock migration in summer, and cultural events that impact fishing [29, 34, 51]. One dummy variable is used per month; it equals one if the observation has occurred in that month, and zero otherwise. Thus, its coefficient measures the impact on the outcome variable that stems from the time in the year when the observation occurs. For example, if a certain cultural event that keeps fishers from harvesting takes place each December, the use of monthly dummies would show a negative coefficient for the December dummy. Moreover, harvest success depends on climate variables, which we assume are entered as an exponential function, in which the exponent comprises linear and quadratic terms of climate variables.

We infer the following results from the estimation. The output elasticity of the capital–labor composite is 0.815 (significant at 1%). The resulting output elasticity of 0.185 for fuel means that fuel expenses constitute 18.5% of total fishing costs. Harvest production also depends on biomass at an estimated elasticity of 0.222 (significant at the 1%-level): harvest increases biomass, but the elasticity is rather small, as should be expected for a schooling fishery. Fish are located more easily at a larger biomass, but as fish form schools, this effect is comparatively weak; once a school is located, a large catch can be made without searching further. We estimate there is a positive linear time trend on catchability at a monthly rate of 0.9% (significant at the 1%-level), which we interpret to reflect technical progress. Furthermore, we estimate monthly dummies and find that catch productivity is highest in spring and winter, and lowest in late summer. Monthly dummies are all significant at least at the 10% level.

To estimate the influence of climate variables on catchability, we test several specifications, including SST, wind speed and direction, as well as precipitation. We refer the reader to Section G of the S1 File for robustness checks. An important determinant of catchability is the SST. We find a non-linear dependence with a peak of 25.4°C at the 1% significance level, a threshold which is usually crossed between May and June. This is in line with the literature, which finds a non-linear dependence and optimal catch temperatures between 21 and 27°C [44, 46, 51]. The stock shifts inshore in spring towards the nursing areas, when upwelling recedes and the temperature rises. The earlier this occurs, the earlier are fish within easy catching distance [32]. When the temperature increases further, *Sardinella aurita* migrates northward and out of immediate reach [34]. This is a natural migration pattern. Interestingly, this feature can explain why we find no negative impact of very high temperatures on biomass growth: As long as it is possible for the species to start procreating earlier in the year, the general increase in SST will not threaten larval survival, considering the fast growth rate of larvae of 2.5/cm per month [29]. No other climate variables were found to have a significant effect on *Sardinella* migration.

According to the model assumptions, fishers minimize costs subject to the prices for capital, labor, and fuel. Capital and labor are supplied on local markets and are not flexibly adaptable following Quaas et al. [78]. Hence, we assume increasing and convex iso-elastic inverse supply functions: in equilibrium, wages and the cost of capital increase with the amount of capital and labor used by the fishery. In previous climate change adaptation studies on agriculture, access to financing has been found to be a major determinant [79]. Fuel supply is perfectly elastic, and prices are determined on the world market. We specify the resulting equilibrium cost function as quadratic, following [80] (see Section G of the S1 File for robustness). Fuel prices already explain a large part of the strong cost trend found in the previous literature [71]. A linear cost time trend is added to include remaining unobserved trends in factor prices and the size of factor units. The trend parameter is estimated to be positive at the 1% level. Net linear time trends in total, ceteris paribus, lead to a monthly increase in fishing pressure of 0.8–1.0%; however, this result is pitted against rising fuel prices, market development, and changing climate conditions. With continued market development, equilibrium harvest becomes more sensitive to input changes, particularly biomass. As consumers become less dependent on fish supply, prices remain stable across large ranges of biomass. The cost-reduction effect of larger biomass becomes more important in relative terms. For the subsequent simulation, we need to model how population density as well as fuel prices develop in the future. Data on population density as described earlier in this section and data on Brent oil prices, available from the U.S. Energy Information Administration [81], are used to estimate linear trends into the future. Thereafter, the trend parameters are assumed to decline linearly over a period of 20 years, as is standard for economic models, such as DICE [82]. For population density, we observe lower growth rates for more dense regions, in line with decreasing UN population projections [83]. We also observe a lower productivity trend rate for the longer time horizon (see Section G of the S1 File) and a decreasing trend in energy prices worldwide.

The important model dynamics from our empirical results are summarized as follows.

Climate enters the economic model in two ways: It affects the growth of the stock and changes the amount of fish that can be harvested sustainably. Higher stock levels decrease harvesting costs. Second, SST has a non-linear, convex impact on harvesting costs, as it affects the spatial distribution of the stock. Thus, an increase in SST can increase the propensity to over-fish.

A higher fuel price leads to a steeper equilibrium cost curve and thus, lower fishing pressure. This is in line with the literature findings that fuel subsidies have led to larger vessels, more powerful engines, longer distances traveled and overall a higher fishing effort [24]. In contrast to capital interest and wages, fuel prices are independent from the fuel quantity used by the fishery. This leaves the fishery particularly sensitive to fuel price changes. An increasing fuel price pushes downwards the curve that defines harvest as a function of the stock and thus, reduces over-fishing. For a varying stock growth curve, this means that high growth years are allowed to cushion against following low growth years. Under low fuel prices, the fishers would immediately catch more, thereby swiftly lowering the biomass. Varying both climate models and fuel price trend sheds light on the interaction between the two, as adaptation takes place. The system is less susceptible to climate impacts under high fuel prices, as they have a relative dampening impact on biomass and SST-induced changes in the cost function. In summary, we expect the elimination of fuel subsidies to be beneficial to the sector.

### 2.3 Climate projections

High uncertainty exists with respect to reanalysis products and bio-geochemistry effects of coastal warming [9, 84–86]. For example, while there is general support among climate models for a continuation in the rise of SST throughout the 21st century, the extent of this rise varies among them [87]. To consider this model uncertainty, we use the output of two different earth system models with two different formulations per model, to investigate the consequences of the model spread for artisanal fisheries. This comparison allows us to analyze the importance of certain traits and differences for the fishery. The use and comparison of different climate models can improve the overall understanding of climate impacts and the variability of outcomes, as weaknesses are partly compensated for and greater reliability is achieved [88, 89]. Two of our projections (ECE-o and ECE-bc) are derived from an extended version of the EC Earth model, based on the model described in [90]. The two other projections (NESM-o and NESM-bc) are based on the Norwegian Earth System Model (NorESM), as described in [91]. ECE-o and NESM-o are the original model versions, whereas ECE-bc and NESM-bc are bias corrected. For ECE-bc, bias correction is achieved through adaptation of the ocean-mixing parameter in the ocean model part. For NESM-bc, bias correction is applied by replacing SST from the ocean model part with anomalies before running the atmospheric model, and vice versa with wind stress. The estimated bias is projected into the future. All models use the representative concentration pathway 8.5 as a basis for radiative forcing, that is, business-as-usual continued forcing.

As is common for climate modeling in the region, all four projections show systematic bias to observed values of SST and wind-stress. We use a standard offline nudging approach [92–94] for each climate scenario and apply constant monthly factors directly to the data. The coefficients are estimated through a regression model to fit the ERA reanalysis data used to estimate the biological and economic model parts. The proportional approach outperforms the approach of adding a constant in our case. The fit is better for SST than for wind stress, as the SST development is far more systematic.

In Section H of the S1 File, we compare the four projections concerning mean, trend, standard deviation, and cyclical behavior of key impacts. On average, both winter and fall upwelling show a slight decrease over time for all four models, and are predicted to be stronger under NESM-o and NESM-bc than under ECE-o and ECE-bc. All mean values for winter upwelling for the period 2014–2079 lie below the average observed value during the estimation period (2001–2013). The upper level of the 95% confidence interval falls below the maximum observed value. For fall upwelling, only NESM-o and NESM-bc averages lie above the estimation period mean. For these two, the 95% confidence interval falls fully within the span of observed values. ECE-o and ECE-bc show substantial variability in inter-annual upwelling.

**Fig 2:**
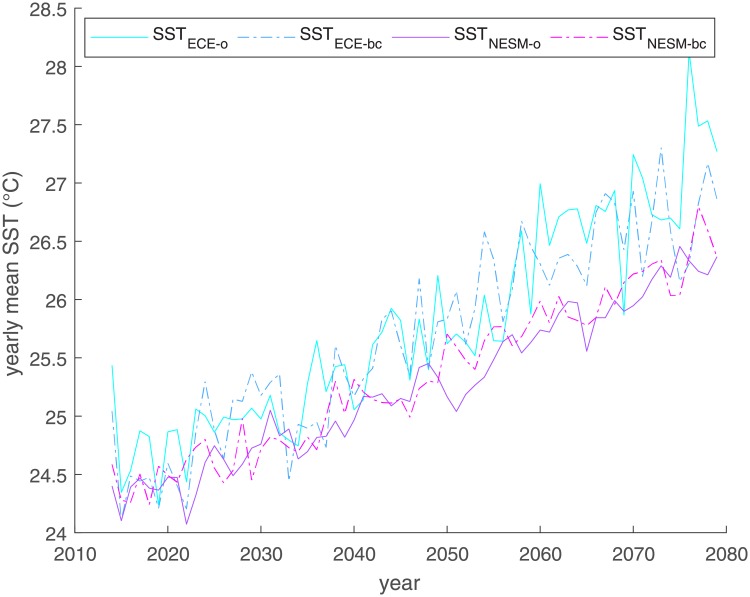
Mean yearly SST over time for different climate models.

A strong upward trend in SST is evident in all four projections (Fig 2). Scenarios ECE-o and ECE-bc feature a higher average temperature than NESM-o and NESM-bc in particular after 2030. They also show a higher variability in yearly means. The difference between warm and cold years is more pronounced. In addition, the seasonal cycle amplitude as well as the pertaining standard deviation are larger under ECE-o and ECE-bc.

### 2.4 Simulation strategy

We run the simulation of the fishery and stock dynamics from 2014 to 2079 or alternatively until the fishery collapses. To account for catches from other, mostly artisanal fleets, we multiply the modeled Senegalese purse seine catches by a factor of 1.083, estimated from historical averages. We use a monthly time step, linearly interpolating fish population growth (which is estimated based on yearly data), including the stochastic term.

**Fig 3:**
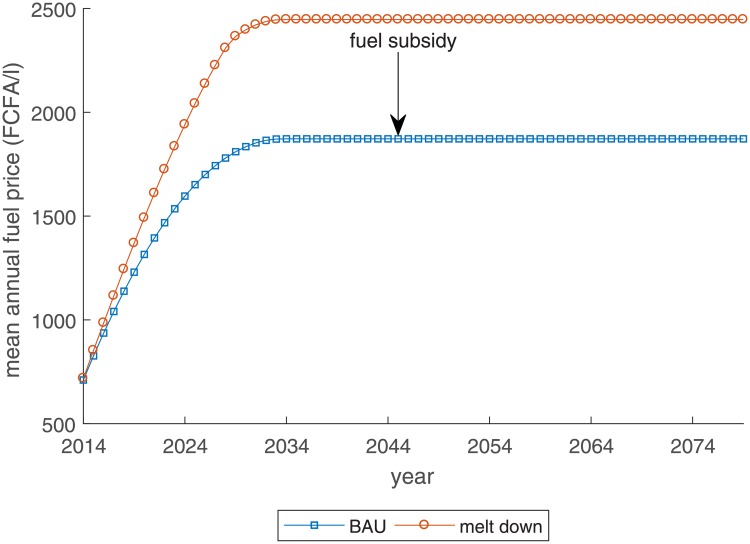
Mean annual fuel price development under different fuel price scenarios.

To evaluate policy alternatives, we consider two scenarios. The development of the mean annual fuel price over time for each of the scenarios is shown in Fig 3. The first is the business-as-usual case (BAU). A proportionate subsidy of 30.7% [95] in the form of tax exemptions has formed the most important component of subsidies [25, 71] since the 1960s. The second scenario considers the case in which the tax-exemption is linearly decreased over 15 years until subsidies are fully eliminated (“melt-down,” md), which enables a gradual transition for fishers and consumers. The price plateaus in 2034, but its level is about 600 FCFA higher under the reform scenario than under the BAU scenario.

In the next section, we first take a closer look at changes in stock growth and the results from spatial stock distribution on catchability. Thereafter, we analyze the model behavior at mean growth and harvest pressure, and more closely consider how the equilibrium between growth and harvest pressure changes over the decades. This allows us to compare the effects of climate projections on the fishery under a stable climate, that is, disregarding short-term variability. We proceed to analyze the dynamic system and focus first on the sustainability of the fishery and the propensity for stock collapse. We then turn to the analysis of welfare effects. Subsequently, we explore the effects of abolishing fuel subsidies on sustainability and welfare.

## 3 Results and discussion

### 3.1 Biological processes and catchability under different climate models

**Fig 4:**
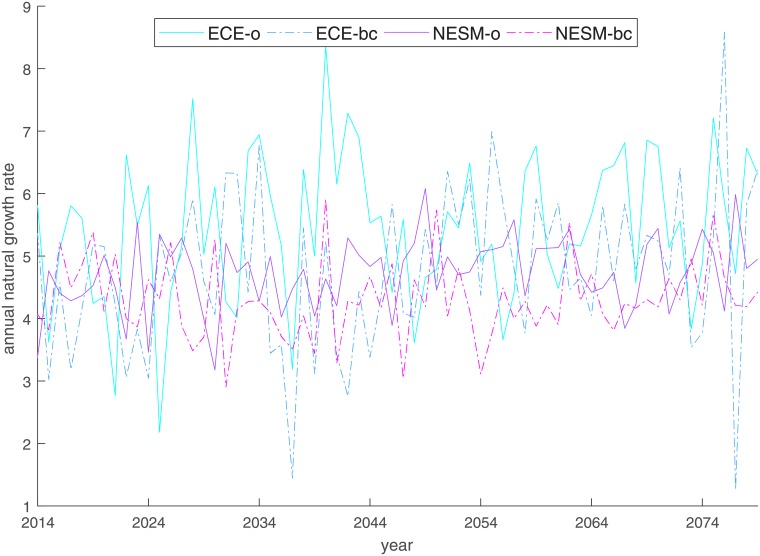
Annual climate affected natural growth rate *r* + *f*(*UP*_*y*_) for different climate projections.

As mentioned in section 2.3, both winter and fall upwelling show a slight decrease over time for all four models, and are predicted to be stronger under NESM-o and NESM-bc than under ECE-o and ECE-bc. The decrease in winter upwelling is beneficial for stock growth, while a decline in fall upwelling is harmful. The overall resulting natural growth rate development is shown in Fig 4. Clearly, its variability is higher under ECE-o and ECE-bc for both seasons, which is in line with the higher upwelling variability. Average system productivity is highest under ECE-o and lowest under NESM-bc, particularly because of the second half of the time horizon. We observe an upward trend for ECE-o and ECE-bc. In the other two projections, the natural growth rate shows no detectable trend, but a distinct dip is visible in the medium term between 2036 and 2057 under ECE-bc.

SST and monthly dummies enter the harvest function in a jointly multiplicative term. Hence, the SST effect on harvest pressure is strongest during the high season between February and May. These months, especially April and May, have particularly high catchability, also due to biological processes uncontrolled by SST. Section I of the S1 File provides a figure showing the development of harvest pressure over time for 4 exemplary months. During the high season, the fishery becomes cheaper over time, as the SST approaches the optimum temperature. The fish remain in the close-to-shore area for a longer period than before, where they are easily caught. Between June and December, the temperature increase leads to a decrease in catchability from 2040 onwards under all climate projections. Fish leave the close-to shore area slightly earlier than before. However, these months are generally less important for the fishery anyway. For both periods, the impact is stronger for ECE-o and ECE-bc, as is the variability. Overall, we observe a severe increase in catchability induced by the SST trend. Toward the end of the time horizon, this trend is either approximately stable (ECE-bc, NESM-o, and NESM-bc) or reversed (ECE-o), when SST already lies above the optimum point during spring. Then, the Senegalese waters finally become too warm for the species, which would permanently relocate northwards. These results are summarized as follows.

**Result 1**. *The impact of upwelling on stock growth is projected to remain largely unchanged from historical values*. *A slight increase is anticipated under ECE-o and ECE-bc*. *The most important climate impact over time is exerted by SST*, *which drives fishing costs down during spring*.

NESM-bc features a comparatively high total harvest pressure over the whole time horizon. At the beginning of the time horizon, yearly harvest pressure is also high under ECE-o, and in the medium term, ECE-bc shows the highest harvest pressure. Under NESM-o, harvest pressure is consistently low.

Our model includes additional factors that impact the harvest curves. Market development provides increasing substitution possibilities for consumers. Consequently, equilibrium harvest becomes more sensitive to the stock level, leading to a steeper harvest function early in the time horizon and a lower equilibrium stock value. In addition, positive trends in fuel prices and technological development combine with a net low positive trend, which strengthens the upward shift in harvest functions until 2033. By our assumptions, these trends dwindle to zero until 2033.

**Result 2**. *In addition to climate impacts*, *the propensity to over-fish is increased by technological development*. *Market development leads to a lower equilibrium stock*. *Increasing fuel prices only insufficiently offset the other impacts*.

### 3.2 Bioeconomic equilibrium analysis

We analyze the model behavior at mean growth and harvest pressure, and take a closer look at how the equilibrium between the two changes over the decades. This allows us to compare the effects of climate projections on the fishery under a stable climate, that is, disregarding short-term variability.

**Fig 5:**
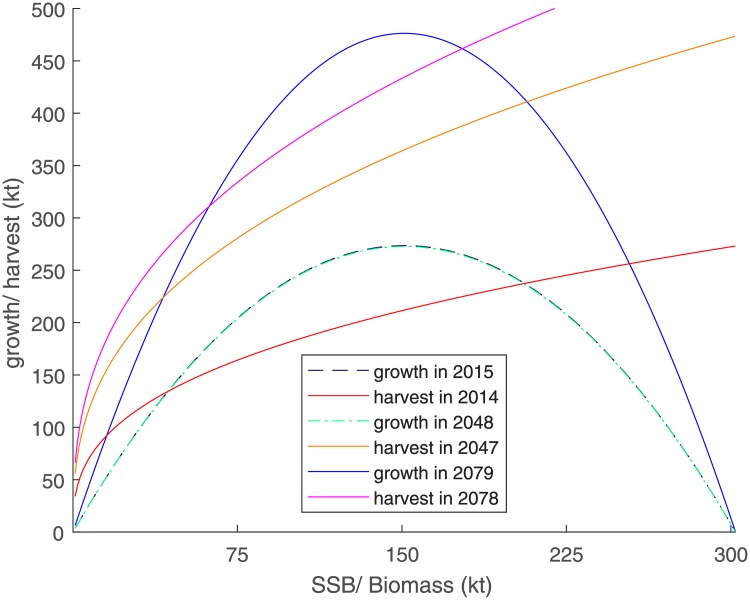
Annual harvest and biomass growth as a function of (spawning stock) biomass for exemplary years under ECE-o.

Fig 5 shows yearly total harvest as a function of biomass, as well as biomass growth, for exemplary years under ECE-o and BAU. We choose growth with a year lead on harvest, because we model growth in year *y* to depend on SSB 1 year before that. The interpretation of the graph is as follows: if the growth function lies above the harvest, the stock shows net growth. In a non-variable, deterministic model, the rightmost interception of the two marks dynamically stable positive equilibrium (see Fig 5) at which the harvest is exactly offset, such that fishers in the next year are able to benefit from stock exactly as large as in the prior year. For harvest years 2013 and 2078, such a point exists. However, for 2047 the harvest curve lies far above the growth curve. The only stable equilibrium in such a graph would be a biomass of zero. If this were the case for longer than a few periods, biomass would collapse in the deterministic case. Note that these are only singular, exemplary years. As a result of climate variability, the shown years cannot be taken as a reference for whole periods.

**Fig 6:**
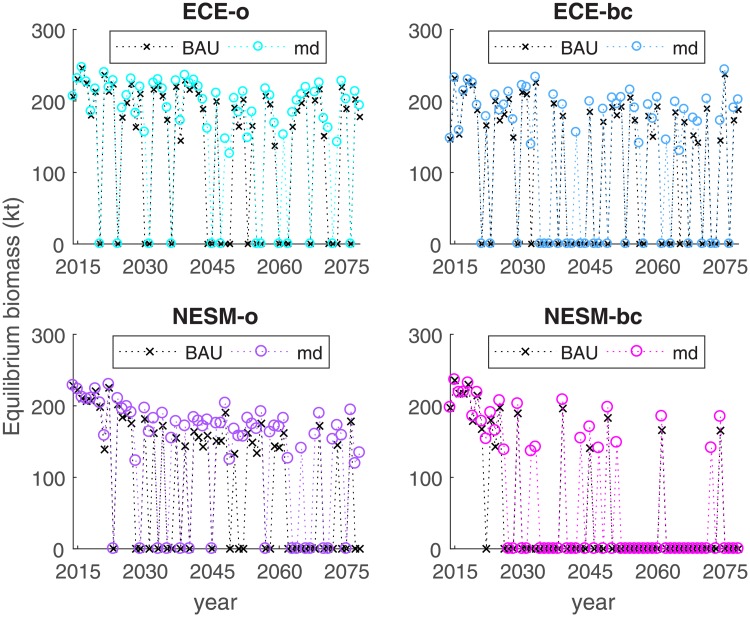
Development of stable equilibrium over time under different climate projections and fuel subsidy scenarios (business as usual, BAU and melt-down, md).

In Fig 6, we show how this equilibrium changes over time for all four climate projections and under the two fuel price scenarios. Consider first the black crosses that indicate the BAU case in which fuel subsidies are maintained. A downward trend in equilibrium biomass is recognizable under all four climate projections. The number of years in which fishing is unsustainable, marked with an equilibrium value of zero, also increases. Under ECE-o, few such years alternate with relatively favorable years and equilibrium biomass around 200 kt. For NESM-bc, after 2028, there are only very few sustainable years for the fishery. After about 2030, a dangerous period arises under NESM-o and ECE-bc, in which over-fishing and extinction are likely. This development ends only late in the time horizon, when the SST is too high throughout the year and the harvest decreases again. In the long term, unsustainable harvesting years prevail for NESM-o, NESM-bc, and ECE-bc.

**Result 3**. *The catch potential increases through higher growth and lowered costs through the SST impact*. *Reduced costs increase future over-fishing*. *Their impact outruns the incipiently favorable climate change effects in three of the four projections under the BAU fuel subsidies*. *The fishery remains sustainable under the ECE-o climate projection*.

If fuel subsidies were (gradually) abolished, harvest pressure would be lower, such that a higher equilibrium biomass would result (see Fig 6). Unsustainable years occur less often, particularly under NESM-o. Ensuring survival under ECE-bc and NESM-bc would prove very difficult in the medium term, when productivity is still relatively low and harvest pressure high, and even under the melt-down scenario, numerous unsustainable years arise.

### 3.3 Stochastic futures: Sustainability under business-as-usual fuel subsidies

The deterministic, average results are subject to additional pressure from climate variability. A particular consequence of variability is greater propensity to collapse. More variable climate conditions, such as under ECE-o and ECE-bc, may lead more often to situations in which stock survival is threatened. This depends particularly on the bio-economic equilibrium biomass around which the actual stock fluctuates driven by the variable climate. If this equilibrium stock size is large, it works as a strong buffer against climatically unfavorable years.

To analyze the systems’ sustainability, we compute the mean time of survival. This standard measure in population viability analysis evaluates stock persistence facing perturbations [96–98]. For each combination of climate projection (4) and fuel subsidy scenario (2), we run the model 1000 times, over which we compute means to capture that the fishery is unequally variable.

**Fig 7:**
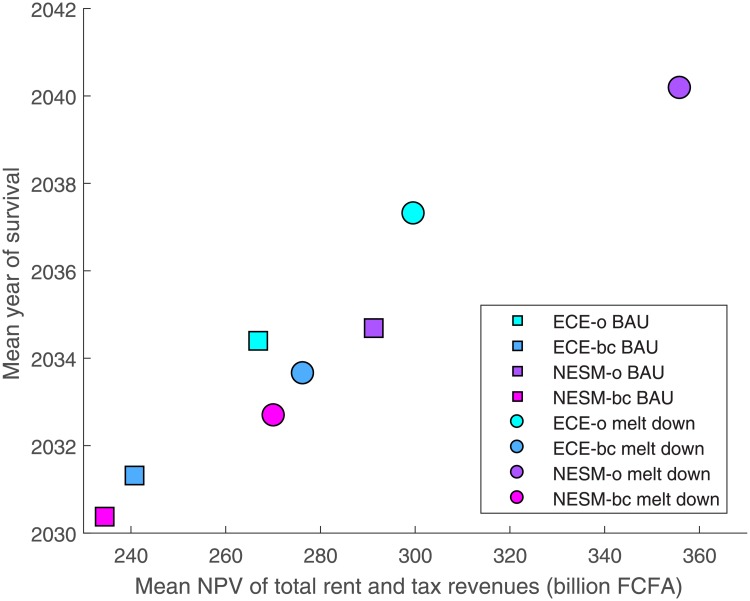
Mean survival horizon and value of the fishery under different climate models and policy scenarios. Colors represent climate projections, shapes represent fuel subsidy scenarios.

Fig 7 shows one scatter plot for eight different models—the combination of four climate projections and two policy scenarios. The mean time of survival is indicated on the y-axis. To compare welfare across climate projections, we use the mean net present value (NPV), indicated on the x-axis. The welfare impact is discussed in the next subsection, 3.4.

The colors indicate the underlying climate projection. The shapes indicate the fuel price scenario. The squares show how a fishery performs under different climate scenarios in the BAU case, in which fuel subsidies are maintained. As expected, variability under ECE-o is so high that the fishery underperforms. The fishery cannot reap the whole benefits of its favorable climate prediction. Short periods of high biomass alternate with long periods of slow replenishment. This dampens mean survival relative to the underlying high productivity. NESM-o becomes steadily more unsustainable in the medium term, leading to extinction around 2035. Under ECE-bc, this effect also plays a role, especially in the early years. Variability is high, which contributes to comparatively low sustainability even before 2030. During the early 2030s, the fishery is particularly vulnerable owing to its low productivity and high harvest pressure. During this time, variability in almost all runs eventually leads to extinction. Under NESM-o, mean survival is in line with the deterministic equilibrium. Variability is too low to have a substantial impact. Between NESM-o and ECE-o, variability is a game changer: NESM-o now outperforms ECE-o, even though the latter shows superior equilibrium sustainability. NESM-bc provides the least favorable climate prediction. Almost from the start, productivity is too low to counter the steadily increasing harvest pressure, and the species becomes extinct early on. Therefore, variability is of little relevance.

**Result 4**. *The BAU scenario is not dynamically sustainable under any of the four climate projections*. *The fishery on average survives only until 2030–2035*. *To ensure greater sustainability*, *resource managers ought to consider policy actions*. *Variability can be a game changer for relative performance under different climate projections*.

We can now also measure climate uncertainty, which we define as the spread in outcomes due to different climate projections. Between NESM-o and NESM-bc, climate uncertainty amounts to 4.5 years of mean survival times, that is, 26% of NESM-bc’s value. The two bias-corrected projections, which were specifically adapted for a better reproduction of the observed climate, are the two with the lowest mean survival time. This indicates that if a study relies only on original biased models, outcomes may be upward biased.

### 3.4 Stochastic futures: Welfare impacts under business-as-usual fuel subsidies

Total sector benefits include the fishery’s role as a safety net, that is, income provision (producer surplus or “fisher surplus,” FS), and its direct contribution to food security (“consumer surplus,” CS). CS is the willingness to pay for fish above the monthly equilibrium price. For our downward-sloping demand curve with price on the y-axis and quantity demanded on the x-axis, CS is the area below the curve and above the horizontal line drawn at the equilibrium price (see Section J of the S1 File). Fishers earn rents because of their role in providing capital and labor to produce harvest. FS can be calculated by summing the areas below the equilibrium price and above the inverse supply curve for capital and labor, respectively. Adding up FS and CS yields a welfare measure for food security, which takes into account the availability of substitutes, and thus, changes in the fishers’ and consumers’ dependence on fresh *Sardinella aurita*.

To compare welfare across climate projections, we use the mean NPV. NPV is a standard economic measure that computes the sum of all future benefits in monetary units and weighs future benefits by a discount factor to capture the higher future value of money that is immediately available. In this study, we use the 2014-NPV of total rents generated in the fishery at an assumed discount rate of 3%. For the scenarios with reduced subsidy, we add the savings of tax revenue. We again compute means over 1000 runs.

We calculate an average FS of 8.77 billion FCFA in 2014 and an average CS of 5.47 billion FCFA. At a typical price of 1,145 FCFA for a 5-kg basket of local grains, this amounts to 946 baskets yearly for each of the roughly 8100 fishers involved, and 1.96 baskets per inhabitant of the coastal region studied (2.4 million, about 21% of the total Senegalese population). These baskets constitute an additional gain for the fishers (consumers) over the situation in which the resource would not be available to them and they would have to work in different sectors (consume different products). Using observed harvest and prices, we calculate a mean yearly total rent value of 14.97 billion FCFA for 2004–2013. This compares well with the summed benefits from 2014, namely, 14.24 billion FCFA. We conclude that our simulation produces plausible results.

In Fig 7, the mean NPV is indicated on the x-axis. Again, we first concentrate on the squares, that is, the BAU case. High variability under ECE-o also has a dampening effect on rents. First, this is a direct consequence of shorter survival times. Second, ECE-o’s survival year profile is right-tailed: extinction is most likely at the beginning of the time horizon, when harvest curves are flattest. The biomass usually becomes extinct early on, and the relatively high sustainability is the consequence of few runs with very long survival. Owing to discounting, the extra rents per occasional additional survival year contribute only a small amount to the NPV. In particular, the yield is lower than under NESM-o, which is less productive, but very stable. Yields are very low under ECE-bc and NESM-bc, that is, the two bias-corrected models. The low productivity in NESM-bc leads to lower potential NPV. Over-fishing and low sustainability in both ECE-bc and NESM-bc also translate to low expected rents.

**Fig 8:**
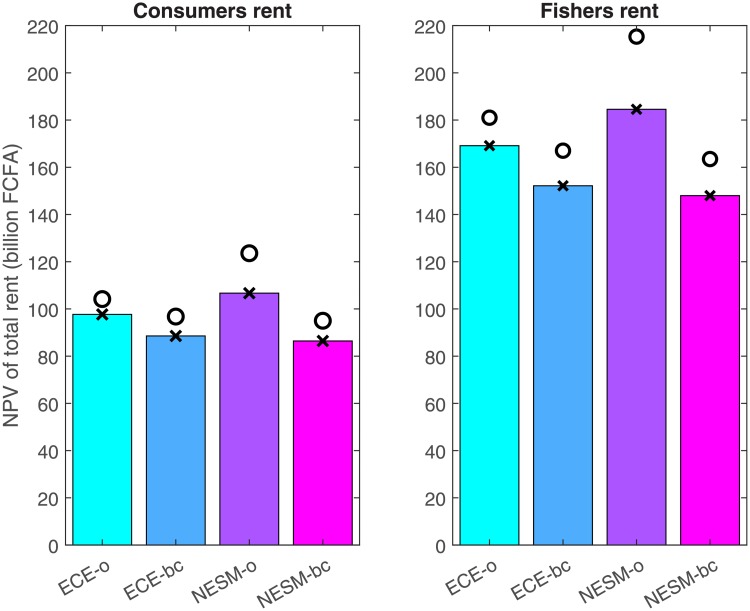
NPV of total rents per model (Bars represent rents under BAU. Shapes represent fuel subsidy scenarios (crosses: BAU, circles: melt-down).

Using the range of total rents as a metric, climate uncertainty amounts to 57 billion FCFA, which constitutes 24% of the lowest NPV of total rents. Fishers’ rent difference accounts for 64% of this gap. We conclude that the effect of climate uncertainty is moderate. This is a direct consequence of low sustainability: short survival times mean that differences in climate projections in the medium term rarely affect rents, because in most cases, the resource by then has gone extinct. Fig 8 shows the distribution of benefits between fishers and consumers for the eight models considered. The effect on income security is larger than on food security. This results mostly because the model predicts that markets develop and consumers’ dependence on fresh *Sardinella aurita* declines. In relative terms, consumers’ mean surplus share is highest under ECE-bc, but the difference across models is exceedingly small.

**Result 5**. *The sector’s contribution to welfare under the BAU case as measured by the mean NPV of FS and CS amounts to 232–291 billion FCFA*. *One-third of this amount can be attributed to a direct contribution from CS*, *and two-thirds to an indirect contribution via the sector’s function as an income safety net*. *The spread between climate projections is moderate at 57 billion FCFA*. *This is a direct consequence of low sustainability*.

To separately analyze changes in annual rents under climate change and to complement the literature findings, we compare undiscounted rents in different decades for each model (see Section K of the S1 File). Climate change is costly under projections ECE-bc and NESM-bc, as well as for the NESM-o BAU scenario owing to actors’ adaptation. [11] suggested that for the whole Senegalese artisanal fishery, 13% of jobs would be lost by 2050. Comparing rents in 2014–2023 and 2034–2043, we observe changes between -97% and 52% under BAU. Mean losses lie at 27% when fuel subsidies continue. This result holds only because of the (small but positive) possibility of very large benefits under ECE-o. On average, the resource would have gone extinct by 2050 in all eight model-scenario combinations considered, such that the potential mean yield for 2054–2063 equals zero under all projections except ECE-o. The risk is substantial.

**Result 6**. *Under three of the four climate projections*, *the sector’s yield potential is reduced in the medium and long terms*. *Until 2043*, *mean rents are reduced by more than one-quarter*.

### 3.5 Welfare impacts if fuel subsidies are abolished

It has been argued in World Trade Organization (WTO) discussions that developing countries’ fisheries may need subsidies to help maintain food security and alleviate poverty [23]. The argument is that fuel subsidies help to increase fishing effort by enabling the use of larger vessel, engines that are more powerful and by allowing fishers to travel larger distances to fishing grounds [24]. The ensuing larger catches lead to lower prices for consumers. The first part corresponds well with our study: Our model predicts that fuel subsidies will lead to more fishing effort. However, once accounting for the dynamic impact of increased fishing pressure on biomass, we find that eliminating fuel subsidies would benefit both fishers and consumers. Higher fuel prices, all else equal, decrease harvest. This reduces over-fishing, such that the equilibrium stock level is higher and more stable. In Fig 7, the circles mark the melt-down subsidy reform case. This would be the outcome if fuel subsidies were gradually decreased and finally fully abolished in 2030. In the most extreme case, NESM-o, this increase reaches 5.4 years mean survival time increase or 62 billion NPV of total rents. It could increase the BAU value by 21%.

Thus, not only are fuel subsidies expensive for taxpayers, they also reduce welfare in total. If fuel subsidies were abolished, climate change might on average even turn out to be slightly beneficial concerning undiscounted rents, that is, the sector’s current value yields (Section K of the S1 File). The subsidies may achieve redistribution of rents from fishers to consumers, but this effect is small and dearly bought with an overall lower consumer rent, as evident in Fig 8.

**Result 7**. *If fuel subsidies were abolished*, *the NPV of total rents would increase regardless of the climate projection*. *Abolishing fuel subsidies would also largely maintain the current distribution of rents between consumers and fishers*.

We quantify this effect to analyze intervention effectiveness. The beneficial effect of removing fuel subsidies is not equally large across climate projections: The rent difference between policy scenarios varies between 33 billion and 62 billion FCFA, that is, 12–21% of the BAU rent value. Intervention effectiveness depends not only on the cost share of fuel for fishers or the size of the intervention. More important is how climate influences the extinction risk. A low stock may result from a system with overall low productivity (e.g. NESM-bc), with SST-induced over-harvesting incentives or from variability, for example after a sequence of unfavorable upwelling years (e.g. ECE-o). If the stock is low, a small difference in catches due to abolished fuel subsidies can be decisive for whether the resource goes extinct or whether it continues to contribute to food security. The benefit of saving the resource from extinction is even larger if the period of unfavorable climate is only temporary and followed by a more favorable climate with a high equilibrium stock and large sustainable catch potential.

Gains due to increased survival time are positive for all scenarios, but largest under NESM-o. Under NESM-o, the abolition of fuel subsidies suffices for the resource to survive for a substantially longer amount of time. For NESM-bc and ECE-bc, policy gains are positive but small, because the intervention is not strong enough for the stock to survive the projected increase in harvest pressure. Under ECE-o, the mean survival year profile becomes more right skewed than in the case with fuel subsidies. This means that the intervention can help to decrease the extinction risk in late years, while it is not sufficient to save the resource in early years (keep in mind that the system is partly stochastic). Due to discounting, these long-run gains have a relatively low weight. Therefore, rent gains per additional survival year are low.

Outcome uncertainty between the different climate projections is increased through fuel subsidy reform. Differences in climate models now translate into larger differences in ensuing rents, that is, up to 84 billion FCFA between the melt-down case under NESM-bc and NESM-o. The increase in uncertainty mostly stems from larger differences in survival times, and longer survival in general such that differences between the climate projections come into effect more and more.

**Result 8**. *Differences in climate projections lead to a spread in the welfare outcome*, *which amounts to more than double the spread between fuel scenarios*.

The main effect of climate change concerns the spatial stock distribution: biomass becomes more vulnerable to Senegalese purse seines over a certain time horizon, because the fish spend more time in the close-to-shore area. Abolishing fuel subsidies could buy time to bridge this transition period. The effectiveness of this measure depends on the climate development. It would be particularly effective under low variability and a weak SST trend, i.e. with a moderate extinction risk. If either variability or the SST trend proved too strong, this measure would be insufficient. A proper resource management system that goes beyond the mere abolition of subsidies, such as a binding limit on annual harvest, would be needed to ensure sustainability in the long term. Nonetheless, there is good news: productivity remains high under all projections except NESM-bc, such that rents and yields remain high if a proper management is installed. However, if the spatial stock redistribution effect crosses national boundaries, such a management would require multinational cooperation.

**Result 9**. *Abolishing fuel subsidies can buy time to counter a dangerous decrease in harvesting costs associated with increased over-fishing*. *A proper resource management system is needed to ensure sustainability in the long term*.

## 4 Conclusion

Our results can inform policymaking, as they shed light on the effect of climate change while incorporating endogenous adaptation of actors. Seemingly beneficial climate impacts, such as a cost reduction due to climate-induced movement of fish stock, may turn out negative, as they increase the propensity to over-fish. When policymakers consider climate change impacts on their fishing sectors, they should not stop at an analysis of catch potential. Even if a fishery currently makes a considerable food and income security contribution under open access and the catch potential would not decrease, climate change might alter the open-access equilibrium to the point where the species is pushed toward extinction. In other words, climate change may alter the need for resource management in a fishery. For the fishery in question, it is clear that without intervention, sustainable resource use is not likely. Moreover, our findings reveal large uncertainty of outcomes with respect to different climate projections, even when qualitative patterns of the climate trajectories are preserved and the differences seem small in the original climate data. Thus, the results underline the need for careful comparative evaluations with different climate projections, and can help policymakers to be on the look-out for important projection traits that may explain outcome differences.

To grasp the severity of climate change impacts fully, it is important to consider complementary future changes, such as those in market development and technological progress. Future market, technology, and factor price development are part of our analysis. Our results show that on aggregate, they accelerate the unsustainable use of the resource. The counteracting increase in fuel prices due to the increase in world market oil prices is not sufficient to turn the tide. Thus, if economic development is neglected in climate change simulation studies, impacts may be underestimated.

We show that abolishing fuel subsidies leads to more sustainable use of the resource. In monetary terms, this is beneficial for the sector as a whole, but also for each of the two factions—consumers and fishers—separately. We conclude that this policy should be considered to enhance overall welfare and to help the sector adapt to climate change. However, this policy alone is not sufficient. It can buy time to implement a proper resource management, such as quotas or—if these are not feasible—more refined effort restrictions. If such a system were successfully implemented, the gains could be large; climate change reduces harvesting costs for a considerable time period. Biomass growth is either unaffected or increases, depending on the climate projection. If over-fishing could be eliminated, the sector would yield a contribution to income and food security that could exceed even current yields. Necessary avenues of future research include the extension of the model to linkages with stocks and fisheries in neighboring Mauritania, as well the inclusion of further fleets and species interactions, once data becomes available. Furthermore, tipping points may play a role: For example, if SST finally becomes too high in nursery areas that can not be compensated by an earlier start of the nursing season, this may threaten larval survival and lead to extinction of the sub-population within a few short years. Further experimental research on such tipping points could shed light on the likelihood of such disastrous events.

## Supporting information

S1 File Appendix. (PDF)
