# Supplementary material for: Climate change adaptation and the role of fuel subsidies: An empirical bio-economic modeling study for an artisanal open-access fishery
Source: PLoS One. 2019 Aug 21;14(8):e0220433. doi: 10.1371/journal.pone.0220433 (PMC6703682; doi:10.1371/journal.pone.0220433)
Supplement: S1 File — (PDF) [file pone.0220433.s001.pdf]

Supporting information: Appendix

A Development of *Sardinella aurita* abundance in Senegalese waters

Fig 9 shows abundance of *Sardinella aurita* in Senegalese and Gambian waters in the fourth quarter of each year, as used in FAO working group stock assessments [35-40]. National surveys provide estimates for 2007–2009 [41-43]. Data points for 2010–2013 are own predictions, as detailed in the main text.

B Use of climate data and calculation of coastal upwelling indicators

For climate impacts, we again make use of the ERA Interim Reanalysis product with monthly time scale. As in the main analysis , the data are provided by the European Centre for Medium-Range Weather Forecasts. We average SST over one 1.5x1.5°coordinate box between 13.5–16.5°N for each of the catch areas for Thiès Sud, Cap Vert/ Thiès Nord, and Fleuve, holding the longitudinal component constant over regions (17.25–18.75°W).

Coastal upwelling indicator: We calculate Ekman transport EK using wind stress (WS) data from quadrants closest to shore but not overlapping with land. We average over the coordinate box between 13.5–16°N and 17.25–18°W. Subscript u denotes the zonal (west–east) component and v the meridional component v:

$$EK_u = \frac{WS_v}{1025\frac{kg}{m^3}2 * sin(lat) * 7.292x10^-5\frac{1}{s}} \quad (1)$$

$$EK_v = -\frac{WS_u}{1025\frac{kg}{m^3}2 * sin(lat) * 7.292x10^-5\frac{1}{s}} \quad (2)$$

Then, we calculate the coastal upwelling indicator (in $\frac{m^3}{s}$ per meter of coastline) as

$$CUI^W = -\sin\left(\varphi - \frac{\pi}{2}\right) EK_u + \cos\left(\varphi - \frac{\pi}{2}\right) EK_v \quad (3)$$

with φ defining the mean angle between the shoreline and equator (90°).

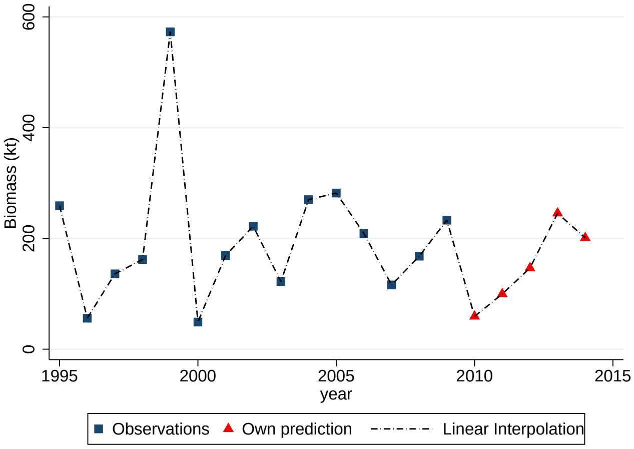

Fig. 9: *Sardinella aurita* abundance estimated by Nansen surveys, national surveys for Senegalese and Gambian waters, and own predictions

SST upwelling indicator: The SST upwelling index (in °C) is defined as the difference between the coastal SST (17.25W) and the SST 5 degrees further toward the open ocean for each latitudinal point (22.5 W):

$$CUI^S = SST_{coast} - SSTocean \tag{4}$$

C Summary of the estimation dataset

A summary of the dataset used in the estimation for both the economic and biological model parts is provided in Table 1.

Table 1: Summary statistics of the dataset used for estimation

	Mean	Sd	Min	Max
Growth estimation (yearly, 1996–2009)				
Spawning stock biomass (kt)	199.50	129.79	49.00	573.00
Total harvest (kt)	181.85	51.57	121.85	272.58
Biomass growth (kt)	179.99	218.89	-402.15	562.19
Windstress CUI (winter) ($\frac{m^3}{s}$ per m coastline)	0.32	0.04	0.25	0.41
Windstress CUI (fall) ($\frac{m^3}{s}$ per m coastline)	0.19	0.03	0.12	0.24
Economic estimation (monthly, 2001–2013)				
Regional Landings (t)	3,768.30	3,583.12	0.90	19,144.76
CPUE (t/DAS)	2.90	2.56	0.00	15.36
Fish price (2010-level FCFA/kg)	131.71	160.41	16.58	2,556.85
Fuel price (2010-level FCFA/l)	485.38	112.26	208.18	731.36
Population density (Number of people/$1000 * \text{km}^2$)	3.34	5.46	0.24	14.02
SST (°C)	24.08	3.00	18.53	28.63

D Biomass growth estimation: Details and robustness

We estimate the effect of environmental variables on yearly stock growth for *Sardinella aurita* found within the Senegalese and Gambian exclusive economic zones, x_y. We use annual biomass growth g_y, defined as the sum of the biomass difference between consecutive years and harvest H_y, as the dependent variable. Biomass growth is positive except in 2000. We interpret the negative value in this year as biomass overshooting its carrying capacity under optimal conditions, which is remedied in the following year. Overshooting of carrying capacity can occur in populations of small pelagic fish with short life span and high recruitment. For species affected by changing environmental conditions, this is to be expected. We model reproduction using the standard logistic growth model with SSB x_{y-1}, carrying capacity K, and natural reproduction rate r.

The growth function is estimated as

$$g_y = x_y - x_{y-1} + H_y = (r + f(UP_y))\ x_{y-1}\left(1 - \frac{x_{y-1}}{K}\right) + \varepsilon^s \qquad (5)$$

where ε^s denotes the error term with normal distribution and standard deviation 80.29 kt. In the simulation, this is entered as a stochastic term. $f(UP_y)$ denotes a linear sum of impacts, in which various structures are tested. We consider alternative specifications with respect to lag structure, seasons, SST, an alternative upwelling index based on SST, and the size of the biomass or biomass growth of the Mauritanian stock group to check for migration flows. We also test alternatives with climate impacts on the carrying capacity, but models with environmental effects on the natural growth rate generally perform better than do models with environmental effects on carrying capacity K, or on the quadratic term in general.

Results are reported in Table Table 2. As a robustness check, we present results for the model without climate impacts (column 1), as well as for the model that provides the best fit with respect to adjusted R^2 and Bayesian information criterion (BIC). The final chosen model is presented in the rightmost column. We include it here for easier comparison. The lower section includes marginal effects at means.

Both models presented below estimate a linear environmental impact on r. Note that when the biomass overshoots the carrying capacity, this means that environmental stressors increase mortality. We explore to what extent the estimation results are driven

Table 2: Estimation results for the biological part of the model

Parameter	model 1	model 2	Chosen model
K	$418.75***$	$290.99***$	$302.13***$
	24.15	13.29	18.22
r	$2.22***$	$-4.06**$	$6.77*$
	0.42	1.51	3.50
$CUI_{W spring,y-1}$		$48.11***$	
		7.01	
$CUI_{SST summer,y-1}$		$-5.05***$	
		1.24	
$CUI_{W winter,y-1}$			$-27.29***$
			6.08
$CUI_{W fall,y-1}$			$30.41**$
			10.55
N	14	14	14
adjusted R^2	0.64	0.92	0.92
AIC	185	165	166
BIC	186	167	168
Variable (mean)	Marginal effects at mean$^+$		
x_{y-1} ($199.5kt$)	0.10.	$-1.39.$	$-1.21.$
$CUI_{W spring,y-1}$ ($4.0\frac{m^3}{s\,100m}$)	..	30.17.	..
$CUI_{SST summer,y-1}$ ($-11.56°\,\frac{C}{10}$)	..	$-31.68.$	..
$CUI_{W winter,y-1}$ ($32.3\frac{m^3}{s\,100m}$)	..	..	$-18.49.$
$CUI_{W fall,y-1}$ ($19.0\frac{m^3}{s\,100m}$)	..	..	20.61.

$^+$One unit change in variable leads to absolute change in growth of ... 1000 t.
* p<0.10, ** p<0.05, *** p<0.01

by the negative observation of biomass growth in 2000. The results when this data point is left out differ only slightly. Upwelling coefficients and carrying capacity become slightly larger. We interpret this as a situation in which the favorable climate conditions also favor the predators' development. The inclusion of environmental variables increases model fit by 28–30 percentage points in adjusted R^2.

The result for the intrinsic growth rate in the pure model $r = 2.22$ is consistent with the results of Pech et al. [99], who estimate $r = 2$, and $K = 117kt$. However, the estimated carrying capacity $K = 419kt$ is > 3 times the size estimated by them. Fig 10 shows that the pure model underestimates growth at lower stock sizes and overestimates growth at larger stock sizes. The model also fails to explain the substantial variation around the basic quadratic function.

In model 2, lagged spring wind-stress CUI and summer SST CUI have a positive and significant impact on biomass growth. Note that the definition of the SST CUI index

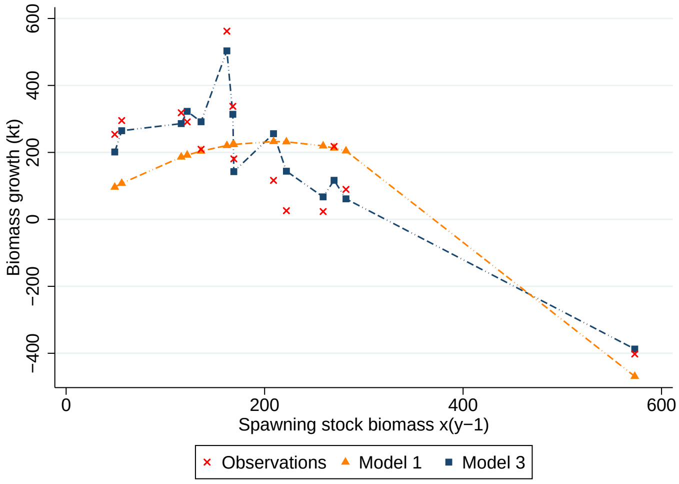

Fig. 10: Growth curves for the Senegambian *Sardinella aurita* stock group, models 1 and 3

Table 3: Predictions of *Sardinella aurita* biomass development for observed harvest levels

		model 1		model 2		model 3	
year	H_y	$\hat{g}_y$	$\hat{x}_y$	$\hat{g}_y$	$\hat{x}_y$	$\hat{g}_y$	$\hat{x}_y$
2010	195.10	229.55	267.45	355.70	393.60	21.28	59.18
2011	224.68	214.62	257.40	-913.07	-744.15	265.21	99.72
2012	211.49	220.28	266.18	-11383.12	-12338.77	258.22	146.45
2013	144.62	215.39	336.95	-2.88E+06	-2.89E+06	243.64	245.46
2014	240.60	146.18	242.55	-6.79E+10	-6.79E+10	196.11	200.98

leads to a negative sign if upwelling is positive and vice versa. We interpret the positive and significant effect on biomass growth as a positive impact on both fall and spring spawning cohorts, as primary productivity is higher. The baseline r is negative: if no upwelling occurs in spring and summer, natural mortality exceeds recruitment. A short simulation to test model performance (see Table 3.) reveals that biomass overshoots the carrying capacity early. Negative growth alone, even when disregarding harvest, then immediately outruns biomass and the stock becomes extinct. We conclude that model 2 is not a good estimate for biomass growth.

Some studies discuss the influence of SST on recruitment success [32, 44, 51, 100]. However, we find no particular impact. The influence of SST likely acts primarily through fish relocation [55]. The Mauritanian stock has a positive and significant influence on the pure growth function (model 1). However, once climate is controlled for, this influence vanishes. Mauritanian biomass growth does not significantly impact

the growth of the Senegalese stock group.

E Economic model: Technical details

Our economic study period runs from January 2001 to December 2013. In addition to yearly biomass data for 2001–2009, we also use our own stock assessment for the years 2010–2013. The general time period t is 1 month. Part of the data is only available per year, denoted by subscript y. We use subscript m to denote dependence on a particular month and subscript i to denote the region.

The harvest is produced from capital k_{it}, labor l_{it}, and fuel f_{it} by the following nested Cobb–Douglas function:

$$H_{it} = q_i(m, W_{it}) x_y^{\chi} \left(l_{it}^{\gamma} k_{it}^{1-\gamma} \right)^{\varphi} f_{it}^{1-\varphi} \tag{6}$$

with $\gamma, \varphi \, \epsilon (0, 1)$. The technology exhibits constant returns to scale. Fishers can substitute between fuel, capital, and labor. Several estimation studies agree that substitution possibilities exist in fisheries, such as [73-77]. We choose a substitution elasticity of one to keep the model simple and tractable. The stock elasticity of biomass [101] $\chi > 0$ is expected to be positive and close to zero, since we model a schooling fishery.

Catchability $q_i(m, W_{it})$ of the fish species depends on the month m and on environmental factors W_{it}. This defines the variability of the species' vulnerability to purse seines, as well as the changes in the vertical and horizontal distribution of the fish. Monthly dummies, denoted by δ_m, represent regular harvest seasonality. We assume that monthly dummies do not vary across regions in order to limit the number of parameters reasonably. For simplicity, we assume that all influences have a direct effect on catchability. In reality, some effects may have influence on factor cost directly. Catchability is assumed to be of the following form:

$$q_i(m, W_{it}) = q_{i0} \, \exp \left(\sum_{m=1, m \neq 9}^{12} (\delta_m \, D_m) + sw_t \, SST + sw_{t2} \, SST^2 \right) \geq 0 \, , \tag{7}$$

where q_{i0} is the reference baseline catchability in September. According to our

estimation, SST has a non-linear, concave influence on catchability, as sw_{t2} is estimated to be negative and small enough. Other weather influences, such as upwelling, precipitation, wind speed, and wind stress, are not found to be significant.

Since data on fuel input are available for the whole fishing sector only, and not for the purse seine fishery in particular, we use a dual approach with fuel prices instead. Fishers use the optimal amount of fuel by equalizing marginal cost, that is, the cost for one unit of fuel Φ_{it}, and marginal revenue, subject to optimally chosen capital and labor amounts.

$$\frac{\partial p_{it} H_i}{\partial f_{it}} = (1-\varphi)\, p_{it} q_i(m, W_{it}) x_y^{\chi} \left(l_{it}^{\gamma} k_{it}^{1-\gamma}\right)^{\varphi} f_{it}^{-\varphi} \overset{!}{=} \Phi_{it} \tag{8}$$

$$\Leftrightarrow f_{it} = \left(\frac{\Phi_{it}}{(1-\varphi) p_{it} q_i(m, W_{it})}\right)^{-\frac{1}{\varphi}} x_y^{\frac{\chi}{\varphi}} l_{it}^{\gamma} k_{it}^{1-\gamma} \tag{9}$$

Reinserting Eq. (9) into the harvest production function, and dividing by the capital–labor composite $l_{it}^{\gamma} k_{it}^{1-\gamma}$, we can define productivity by the following catch per unit of the capital–labor composite ($CPUb_{it}$) function:

$$h_{it} = \left((1-\varphi)^{1-\varphi} q_i(m, W_{it}) \left(\frac{p_{it}}{\Phi_{it}}\right)^{1-\varphi} x_y^{\chi}\right)^{\frac{1}{\varphi}} l_{it}^{\gamma} k_{it}^{1-\gamma} \tag{10}$$

$$\Leftrightarrow CPUb_{it} = \left((1-\varphi)^{1-\varphi} q_i(m, W_{it}) \left(\frac{p_{it}}{\Phi_{it}}\right)^{1-\varphi} x_y^{\chi}\right)^{\frac{1}{\varphi}} \tag{11}$$

This expression is used for the estimation. However, to estimate the full system, we need two additional equations concerning harvest and prices. To derive these, we start with the derivation of the cost function.

Fishers minimize total cost. They are price takers with respect to factor prices. The Lagrangian optimization problem and the first-order conditions for this minimization

problem are

$$L = w_{it}l_{it} + r_{it}k_{it} + \Phi_{it}f_{it} - \rho\left(q_i(m,W_{it})x_y^{\chi}\left(l_{it}^{\gamma}k_{it}^{1-\gamma}\right)^{\varphi}f^{1-\varphi} - \bar{H}\right) \tag{12}$$

$$\Phi_{it} = \rho q_i(m,W_{it})x_y^{\chi}\left(1-\varphi\right)\left(l_{it}^{\gamma}k_{it}^{1-\gamma}\right)^{\varphi}f^{-\varphi} \tag{13}$$

$$w_{it} = \rho q_i(m,W_{it})x_y^{\chi}\varphi\gamma\left(l_{it}^{\gamma}k_{it}^{1-\gamma}\right)^{\varphi}f^{1-\varphi}\frac{1}{l_{it}} \tag{14}$$

$$r_{it} = \rho q_i(m,W_{it})x_y^{\chi}\varphi\left(1-\gamma\right)\left(l_{it}^{\gamma}k_{it}^{1-\gamma}\right)^{\varphi}f^{1-\varphi}\frac{1}{k_{it}} \tag{15}$$

We derive the following factor demand expressions for fuel, capital, and labor from the first-order conditions:

$$k_{it}^{D} = \frac{h_{it}}{q_i(m,W_{it})x_y^{\chi}}\left(\frac{r_{it}}{(1-\gamma)}\right)^{\varphi(1-\gamma)-1}\left(\frac{w_{it}}{\gamma}\right)^{\varphi\gamma}\left(\Phi_{it}\frac{(\varphi)}{1-\varphi}\right)^{1-\varphi} \tag{16}$$

$$l_{it}^{D} = \gamma\frac{r_{it}k_{it}}{w_{it}\left(1-\gamma\right)} = \frac{h_{it}}{q_i(m,W_{it})x_y^{\chi}}\left(\frac{r_{it}}{(1-\gamma)}\right)^{\varphi(1-\gamma)}\left(\frac{w_{it}}{\gamma}\right)^{\varphi\gamma-1}\left(\Phi_{it}\frac{(\varphi)}{1-\varphi}\right)^{1-\varphi} \tag{17}$$

$$f_{it}^{D} = \frac{h_{it}}{q_i(m,W_{it})x_y^{\chi}}\left(\frac{r_{it}}{(1-\gamma)}\right)^{-\varphi(\gamma-1)}\left(\frac{w_{it}}{\gamma}\right)^{\varphi\gamma}\left(\frac{(\varphi)}{1-\varphi}\right)^{-\varphi}\Phi_{it}^{-\varphi} \tag{18}$$

Inserting factor demand into $C = w_{it}l_{it} + r_{it}k_{it} + \Phi_{it}f_{it}$ leads to a cost function that is linear in harvest and decreases in biomass:

$$C = \frac{h_{it}}{q_i(m,W_{it})x_y^{\chi}}\left(\frac{r_{it}}{(1-\gamma)}\right)^{\varphi(1-\gamma)}\left(\frac{w_{it}}{\gamma}\right)^{\varphi\gamma}\left(\Phi_{it}\frac{(\varphi)}{1-\varphi}\right)^{1-\varphi}\frac{1}{\varphi} \tag{19}$$

Thus, marginal cost equals average cost in terms of the fishers' supply decision. Capital and labor inverse supply functions are assumed to be upward sloping [78]. This reflects the localized nature of markets for these two factors, as well as the imperfect or inflexible adaptability of both inputs. Workers obtain a particular skill set to work in the fishery. Capital is invested into a particular type of boat and gear. These two processes are not easily reversed, such that at low demand, supply prices are low. However, they may become increasingly steep as factor demand increases. Then, the supply prices for capital and labor form an increasing and convex function of the respective factor demand. We assume the following iso-elastic inverse supply functions for labor and capital:

$$w_{it}(l_{it}) = \lambda_i \, l_{it}^{\psi}, \tag{20}$$

$$r_{it}(k_{it}) = \kappa_i \, k_{it}^{\zeta}, \tag{21}$$

respectively. Meanwhile, fuel supply is perfectly inelastic at price Φ_{it}. The reason is that fuel prices are exogenous to the regional Senegalese market, and follow world market prices for crude oil. We derive symmetric equilibrium factor prices for labor and capital using factor demand from (16)–(18) in (20) and in (21):

$$w_{it} = \left(\left(\kappa_i \, (1-\gamma)^{\zeta} \right)^{\varphi(1-\gamma)\psi} \left(\frac{h_{it}}{q_i(m, W_{it}) x_y^{\chi}} M \Phi_{it}^{1-\varphi} \right)^{\psi(\zeta+1)} \left(\lambda_i \gamma^{\psi} \right)^{1+\zeta-\zeta\varphi(1-\gamma)} \right)^{\frac{1}{(1+\psi)(\zeta+1-\zeta\varphi(1-\gamma))-\psi\varphi\gamma(\zeta+1)}} \tag{22}$$

$$r_{it} = \left(\left(\kappa_i \, (1-\gamma)^{\zeta} \right)^{1+\psi-\psi\varphi\gamma} \left(\frac{h_{it}}{q_i(m, W_{it}) x_y^{\chi}} M \Phi_{it}^{1-\varphi} \right)^{\zeta(1+\psi)} \left(\lambda_i \gamma^{\psi} \right)^{\gamma\varphi\zeta} \right)^{\frac{1}{(1+\psi)(\zeta+1-\zeta\varphi(1-\gamma))-\psi\varphi\gamma(\zeta+1)}} \tag{23}$$

with

$$M := \left(\tfrac{1}{(1-\gamma)} \right)^{\varphi(1-\gamma)} \left(\tfrac{1}{\gamma} \right)^{\varphi\gamma} \left(\tfrac{(\varphi)}{1-\varphi} \right)^{1-\varphi}$$

Inserting equilibrium factor prices into the cost function leads to the equilibrium cost function, which is non-linear in equilibrium harvest:

$$C = \frac{c_i}{e+1} \left(\frac{h_{it}}{q_i(m, W_{it}) \, (1-\varphi)^{1-\varphi} \, x_y^{\chi}} \Phi_{it}^{1-\varphi} \right)^{e+1}$$

With cost elasticity parameter e and region-specific scaling parameter $c_i > 0$,

$$e = \frac{(1-\gamma)\,\zeta\varphi + (\zeta+\gamma)\,\varphi\psi}{(1+\psi)\,(\zeta+1-\zeta\varphi\,(1-\gamma)) - \psi\varphi\gamma\,(\zeta+1)}$$

$$c_i = \left(\left(\frac{1}{\varphi} \right)^{1+\gamma\zeta+(1-\gamma)\psi} \left(\frac{\kappa_i}{1-\gamma} \right)^{(1-\gamma)(1+\psi)} \left(\frac{\lambda_i}{\gamma} \right)^{\gamma(\zeta+1)} \right)^{\frac{\varphi}{(1+\psi)(\zeta+1-\zeta\varphi(1-\gamma))-\psi\varphi\gamma(\zeta+1)}} \quad (e+1)$$

We fix the cost elasticity parameter $e = 1$, thereby specifying a quadratic cost function, following [80].

Inverse demand is an iso-elastic, decreasing function of the harvest. Our estimation results show that the inverse price elasticity ν depends negatively on regional

population density $B_{i\,y}$, which we interpret as a proxy for the availability of substitutesẆe use density instead of total population in order to account for the differences in market area size. Then, inverse demand is given by

$$p_{it} = \bar{p_i} h_{it}^{-\nu(B_{i\,y})} = \bar{p_i} h_{it}^{-exp(\nu_0 + \eta\, B_{i\,y})} \tag{24}$$

with inverse price elasticity $0 \leq \nu(B_{i\,y}) < 1$.

F Estimation model and estimation results

To estimate supply and demand simultaneously, we derive the reduced-form equations. The fishery operates under open access, such that profits are driven to zero in equilibrium. We set marginal equilibrium cost equal to the equilibrium price. Then, the equilibrium harvest level is

$$h_{it} = \left(\frac{\bar{p_i}}{c_i} \left(q_i(m, W_{it})\,(1-\varphi)^{1-\varphi}\, x_y^{\chi} \Phi_{it}^{-(1-\varphi)} \right)^{e+1} \right)^{\frac{1}{(\nu+e)}} \tag{25}$$

Harvest pressure M, as referenced in Section 2.3 in the main text, is defined as the factor in front of biomass in (25), summed over all four regions:

$$M := \sum_{i=1}^{4} \left[\left(\frac{\bar{p_i}}{c_i} \left(q_i(m, W_{it})\,(1-\varphi)^{1-\varphi}\, \Phi_{it}^{-(1-\varphi)} \right)^{e+1} \right)^{\frac{1}{(\nu+e)}} \right] \tag{26}$$

Inserting (25) back into (24) leads to the following reduced-form expression for the equilibrium price:

$$p_{it} = \bar{p_i}^{\frac{e}{(\nu+e)}} c_i^{\frac{\nu}{\nu+e}} \left(q_i(m, W_{it})\,(1-\varphi)^{1-\varphi}\, x_y^{\chi} \Phi_{it}^{-(1-\varphi)} \right)^{-\frac{\nu(e+1)}{\nu+e}} \tag{27}$$

To achieve full identification, we use harvest productivity in our estimation. The fish price in (11) is substituted by its reduced-form function, such that

$$CPUb_{it} \quad = (\bar{p_i}^{e} c_i^{\nu})^{\frac{1-\varphi}{\varphi(e+\nu)}} \left(q_i(m, W_{it})\,(1-\varphi)^{1-\varphi}) x_y^{\chi} \Phi_{it}^{-(1-\varphi)} \right)^{\frac{e(1-\nu)+\nu\varphi(1+e)}{\varphi(e+\nu)}} \tag{28}$$

We introduce a linear time trend τ_q to capture technical progress in the fishery that is unobserved, since we lack data on boat and motor size as well as other technical characteristics. We add a linear time trend on c_i to include unobserved trends in factor prices and size of factor units. A time trend on $\bar{p_i}$ is found to be significant for the long-term estimation. All time trends are assumed equal across regions. Equal trends are expected at least in the long term, as regions interact and converge. Our estimation shows that this is adequate, and that a linear trend is superior to a convex trend in the long term. Taking logs, including trends and adding an error terms provides the full system of three reduced-form equations to be jointly estimated:

$$\begin{aligned}
\ln p_{it} &= \tfrac{\nu}{(\nu+e)} \left(\tfrac{e}{\nu} \left(\ln \bar{p_i} + \ln(1+\tau_p(t-t_0))\right) + \ln c_i + \ln(1+\tau_c(t-t_0)) - (e+1)HR \right) \\
&\quad + \epsilon_{ht} \qquad (29) \\
\ln h_{it} &= \tfrac{1}{(\nu+e)} \left(\ln \bar{p_i} + \ln(1+\tau_p(t-t_0)) - \ln c_i - \ln(1+\tau_c(t-t_0)) + (e+1)HR\right) \\
&\quad + \epsilon_{ht} \qquad (30) \\
\ln CPUb_{it} = &\ \tfrac{1-\varphi}{\varphi(e+\nu)} \left(e \left(\ln \bar{p_i} + \ln(1+\tau_p(t-t_0)\right) + \nu \left(\ln c \ln(1+\tau_c(t-t_0))\right)\right) + \tfrac{e(1-\nu)+\nu\varphi(1+e)}{\varphi(e+\nu)} HR \\
&\quad + \epsilon_{ct} \qquad (31)
\end{aligned}$$

with

$$HR := \ln q_i(m, W_{it}) + (1-\varphi)\ln(1-\varphi) + \chi \ln x_y + \ln(1+\tau_q(t-t_0)) - (1-\varphi)\Phi_{it} \quad (32)$$

The model is estimated using iterated feasible generalized least squares. The results are reported in Table Table 4 and diagnostic statistics listed in Table Table 5. Note that for simplicity, the reported $\ln Q_{0i}$ is a composite term, where $Q_{0i} := q_{0i}\,(1-\varphi)^{1-\varphi}$. We choose between models with different weather impacts by means of the Akaike and Bayesian information criteria (AIC and BIC, respectively) and model fit. This leads to a specification in which only SST influences catchability, which is included as the non-linear specification $sw_t SST + sw_{t2} SST^2$. We employ robust standard errors, since heteroscedasticity is present in the harvest equation. Visual inspection and augmented Dickey–Fuller tests reveal a trend and seasonality, as expected, but the null hypothesis

of a unit-root when accounting for the trend cannot be rejected. Test statistics for all regions are available upon request. The inverse demand price elasticity depends negatively on population density. For Thiès Sud, Thiès Nord, and Fleuve, this elasticity lies between 0.252 and 0.339. For Cap Vert, $\hat{\nu}$ goes to zero, and prices are independent of quantities.

Table 4: Main estimation results economy

	Coeff./std. error	
χ	0.222∗∗∗	(0.073)
φ	0.815∗∗∗	(0.051)
$\ln Q_{0FL}$	−17.246∗∗∗	(4.537)
$\ln Q_{0TN}$	−18.102∗∗∗	(4.528)
$\ln Q_{0CV}$	−18.620∗∗∗	(4.549)
$\ln Q_{0TS}$	−17.582∗∗∗	(4.532)
δ_1	1.179∗∗∗	(0.348)
δ_2	1.175∗∗∗	(0.370)
δ_3	1.397∗∗∗	(0.366)
δ_4	1.508∗∗∗	(0.361)
δ_5	1.567∗∗∗	(0.345)
δ_6	1.305∗∗∗	(0.280)
δ_7	0.529∗∗	(0.235)
δ_8	0.428∗∗	(0.212)
δ_{10}	0.370∗	(0.200)
δ_{11}	0.856∗∗∗	(0.203)
δ_{12}	1.263∗∗∗	(0.278)
sw_t	1.276∗∗∗	(0.405)
sw_{t2}	−0.025∗∗∗	(0.009)
τ_q	0.009∗∗∗	(0.002)
ν_0	−0.640∗∗∗	(0.216)
η	−1.862∗∗∗	(0.618)
$\ln \bar{p}_{FL}$	6.968∗∗∗	(0.356)
$\ln \bar{p}_{TN}$	6.570∗∗∗	(0.311)
$\ln \bar{p}_{CV}$	4.702∗∗∗	(0.113)
$\ln \bar{p}_{TS}$	6.276∗∗∗	(0.351)
τ_p	0.002∗	(0.001)
$\ln c_{FL}$	−3.127∗∗∗	(0.544)
$\ln c_{TN}$	−4.018∗∗∗	(0.399)
$\ln c_{CV}$	−5.003∗∗∗	(0.375)
$\ln c_{TS}$	−5.138∗∗∗	(0.389)
τ_c	0.010∗∗∗	(0.004)

* p<0.10, ** p<0.05, *** p<0.01

G Estimation of the economic part: Robustness checks

This part provides evidence of the robustness of our economic estimation results with respect to cost elasticity, the use of our own stock assessment biomass estimates, as well

Table 5: Statistics for main estimation results economy

Stat.	Value
N	561
No. variables	32
R^2 eq. price	.34
R^2 eq. harvest	.32
R^2 eq. CPUE	.32
AIC	3917
BIC	4056

as the use of North sea crude oil prices as an instrument for fuel prices. The results are reported in Tables Table 6 and Table 7, with regions denoted by TS for Thiès Sud, FL for Fleuve, CV for Cap Vert, and TN for Thiès Nord.

The cost function is assumed to be largely quadratic in section E. The first two columns show that the results remain robust for lower and higher values of cost elasticity. The first alternative, $e + 1 = 1.8$, performs slightly worse with respect to the Bayesian criterion. Stock elasticity $\hat{\chi}$ and boat production elasticity $\hat{\varphi}$ are lower. For $e + 1 = 2.2$, the results are very similar to the main estimation, except for a slightly higher $\hat{\chi}$. The cost parameters start slightly lower, with a monthly trend of 1.3%. Consequently, the mean positive net trend on equilibrium harvest is reduced to 0.7–0.9% here.

Column 3 in Table Table 6 shows that most results remain robust if only the biomass estimates from the original dataset are used, that is, 2001–2009. Stock elasticity $\hat{\chi}$ is now slightly lower at 0.186. Catchability still depends on SST, but the effect is no longer significantly non-linear. This combines with relatively higher baseline catchabilities and a stronger trend on catchability. The ensuing mean $\hat{\nu}$ is still close to zero for Cap Vert and between 0.385 and 0.436 for the other regions. The net trend in equilibrium harvest increases to 3.21–4.57%. We conclude that the stock assessment results are plausible and compare well with the results gained only from the original biomass data. Not surprisingly, trends are sensitive to a shortening of the time period analyzed, probably because they are not perfectly constant and depend on technological breakthroughs and economic cycles.

Considering robustness with regard to fuel prices, we instrument them using North sea crude oil prices for the Brent type (columns 4 and 5). For the short time period, results are similar to those obtained with the regional fuel prices. However, for the time

horizon that includes the years 2010–2013, the results are not plausible, particularly $\hat{\varphi} > 1$. However, there is a noticeable decoupling of the two price time series: while correlation of logarithmized prices for regional fuel and Brent is at 84% for 2001–2009, there is correlation of only 49% for 2010–2013. We consider that the fact that regional price movements are apparently important should not be regarded as a problem for endogeneity. It is clear that at a yearly mean fuel use of only 50,322,000 l for the whole artisanal fishery, the purse-seine sub-sector is too small to have a notable influence on fuel prices. Indeed, if the regional fuel price is indeed a relevant factor, we would not expect robust results for a time period in which Brent and regional fuel prices develop in opposite directions.

Table 6: Robustness results: Harvest productivity

Parameter	$e = 0.8$	$e = 1.2$	Short time horizon	Short/ brent prices	Long/ brent prices
χ	0.202***(0.068)	0.239***(0.078)	0.186* (0.095)	0.178* (0.095)	0.186***(0.069)
ϕ	0.724***(0.045)	0.885***(0.054)	0.734***(0.044)	0.771***(0.047)	1.777***(0.108)
$\ln Q_{0FL}$	−16.287***(4.187)	−18.020***(4.819)	−12.583***(4.403)	−12.363***(4.372)	−11.408***(4.142)
$\ln Q_{0TN}$	−17.039***(4.178)	−18.956***(4.811)	−13.276***(4.387)	−13.087***(4.355)	−13.300***(4.127)
$\ln Q_{0CV}$	−17.527***(4.197)	−19.497***(4.833)	−13.705***(4.416)	−13.523***(4.386)	−14.165***(4.158)
$\ln Q_{0TS}$	−16.509***(4.182)	−18.443***(4.815)	−12.908***(4.400)	−12.734***(4.369)	−12.928***(4.136)
δ_1	1.048***(0.323)	1.288***(0.368)	1.932***(0.373)	1.958***(0.372)	1.004***(0.333)
δ_2	1.052***(0.343)	1.279***(0.392)	1.898***(0.397)	1.939***(0.393)	0.810** (0.364)
δ_3	1.259***(0.339)	1.514***(0.387)	2.124***(0.387)	2.143***(0.384)	1.049***(0.360)
δ_4	1.360***(0.335)	1.633***(0.382)	2.285***(0.377)	2.291***(0.375)	1.192***(0.353)
δ_5	1.415***(0.320)	1.692***(0.365)	2.199***(0.359)	2.195***(0.359)	1.436***(0.332)
δ_6	1.173***(0.260)	1.414***(0.297)	1.964***(0.295)	1.966***(0.294)	1.333***(0.266)
δ_7	0.467** (0.221)	0.581** (0.247)	0.868***(0.234)	0.875***(0.234)	0.626***(0.196)
δ_8	0.385* (0.198)	0.463** (0.224)	0.653***(0.189)	0.652***(0.195)	0.496***(0.189)
δ_{10}	0.334* (0.187)	0.398* (0.210)	0.235 (0.179)	0.271 (0.177)	0.387** (0.170)
δ_{11}	0.774***(0.188)	0.923***(0.215)	1.130***(0.197)	1.166***(0.198)	0.766***(0.185)
δ_{12}	1.133***(0.258)	1.370***(0.294)	1.730***(0.295)	1.768***(0.292)	1.010***(0.283)
sw_t	1.226***(0.374)	1.314***(0.429)	0.709* (0.392)	0.639* (0.388)	1.055***(0.369)
sw_{t2}	−0.024***(0.008)	−0.026***(0.009)	−0.011 (0.009)	−0.010 (0.008)	−0.021***(0.008)
τ_q	0.008***(0.002)	0.010***(0.003)	0.028***(0.006)	0.038***(0.008)	−0.005***(0.000)

* p<0.10, ** p<0.05, *** p<0.01, Std. errors in parentheses

Table 7: Robustness results: Markets and statistics

Parameter	$e = 0.8$	$e = 1.2$	Short time horizon	Short/ brent prices	Long/ brent prices
ν_0	−0.690∗∗∗(0.218)	−0.603∗∗∗(0.214)	−0.571∗∗ (0.264)	−0.604∗∗ (0.264)	−0.712∗∗∗(0.108)
η	−1.737∗∗∗(0.603)	−1.951∗∗∗(0.626)	−1.087 (0.795)	−1.005 (0.783)	−0.000 (0.011)
$\ln \bar{p}_{FL}$	6.924∗∗∗(0.359)	7.004∗∗∗(0.354)	7.992∗∗∗(0.495)	7.963∗∗∗(0.490)	8.476∗∗∗(0.440)
$\ln \bar{p}_{TN}$	6.533∗∗∗(0.314)	6.600∗∗∗(0.309)	7.538∗∗∗(0.425)	7.517∗∗∗(0.420)	7.981∗∗∗(0.381)
$\ln \bar{p}_{CV}$	4.693∗∗∗(0.112)	4.710∗∗∗(0.114)	4.866∗∗∗(0.121)	4.872∗∗∗(0.119)	8.259∗∗∗(0.393)
$\ln \bar{p}_{TS}$	6.246∗∗∗(0.355)	6.301∗∗∗(0.348)	7.259∗∗∗(0.483)	7.241∗∗∗(0.476)	8.125∗∗∗(0.454)
τ_p	0.002∗ (0.001)	0.002∗ (0.001)	−0.002 (0.001)	−0.002 (0.001)	0.001 (0.001)
$\ln c_{FL}$	−2.486∗∗∗(0.441)	−3.816∗∗∗(0.635)	−4.204∗∗∗(0.492)	−3.801∗∗∗(0.522)	8.886∗∗∗(1.173)
$\ln c_{TN}$	−3.198∗∗∗(0.324)	−4.876∗∗∗(0.463)	−4.674∗∗∗(0.386)	−4.344∗∗∗(0.414)	5.890∗∗∗(0.960)
$\ln c_{CV}$	−3.988∗∗∗(0.305)	−6.054∗∗∗(0.436)	−5.390∗∗∗(0.380)	−5.075∗∗∗(0.401)	4.142∗∗∗(0.882)
$\ln c_{TS}$	−4.095∗∗∗(0.315)	−6.218∗∗∗(0.452)	−5.803∗∗∗(0.355)	−5.503∗∗∗(0.383)	4.479∗∗∗(0.924)
τ_c	0.007∗∗∗(0.002)	0.013∗∗ (0.006)	0.008∗∗∗(0.003)	0.009∗∗∗(0.003)	0.002 (0.004)
N	561	561	374	374	561
No. variables	32	32	32	32	32
R^2 eq. price	0.34	0.34	0.38	0.38	0.36
R^2 eq. harvest	0.31	0.31	0.33	0.33	0.29
R^2 eq. CPUE	0.32	0.32	0.38	0.37	0.31
AIC	3921	3915	2408	2412	3914
BIC	4060	4053	2534	2538	4053

* p<0.10, ** p<0.05, *** p<0.01, Std. errors in parentheses

H Climate model characteristics

The following Table 8 shows statistics that characterize SST and wind stress development for the four climate models used in the simulation. Fig 2 in the main text shows the development of annual mean SST over time for the four climate models.

On average, both winter and fall upwelling is predicted to be stronger under NESM-o and NESM-bc than under ECE-o and ECE-bc. Winter upwelling as well as fall upwelling show a slight decrease over time for all four models. Clearly, the variability is higher under ECE-o and ECE-bc for both seasons.

Table 8: Climate model statistics

	ECE-o	ECE-bc	NESM-o	NESM-bc
SST(°C)				
Mean (2014-2079)	25.778	25.680	25.246	25.334
Mean (2014-2023)	24.750	24.482	24.346	24.473
Mean (2070-2079)	27.141	26.700	26.224	26.331
Standard deviation	3.374	3.333	3.100	3.056
95% confidence interval, upper	32.391	32.222	31.322	31.324
95% confidence interval, lower	19.165	19.156	19.170	19.344
Mean seasonal amplitude	9.339	9.269	8.125	8.004
Std. deviation seasonal amplitude	0.937	0.714	0.282	0.251
$CUI_{winter}(\frac{m^3}{s}$ per m coastline)				
Mean (2014-2079)	0.232	0.255	0.311	0.319
Mean (2014-2023)	0.257	0.288	0.327	0.323
Mean (2070-2079)	0.206	0.240	0.299	0.306
Standard deviation	0.032	0.053	0.020	0.013
95% confidence interval, upper	0.295	0.359	0.350	0.344
95% confidence interval, lower	0.169	0.151	0.272	0.294
$CUI_{fall}(\frac{m^3}{s}$ per m coastline)				
Mean (2014-2079)	0.165	0.163	0.213	0.205
Mean (2014-2023)	0.172	0.165	0.218	0.216
Mean (2070-2079)	0.150	0.163	0.208	0.201
Standard deviation	0.0274	0.026	0.011	0.014
95% confidence interval, upper	0.219	0.214	0.235	0.232
95% confidence interval, lower	0.111	0.112	0.191	0.178

I Harvest pressure over time

We call harvest pressure M the multiplier in front of biomass in equilibrium harvest (see F). It depends on SST, fuel prices, monthly dummies, the change in the inverse price elasticity via population growth, and time trends. Fig 11 shows the development over

time for 4 exemplary months. We choose May, June, December, and January, because they constitute the transition months with temperatures often around the optimum temperature, 25.4° C.

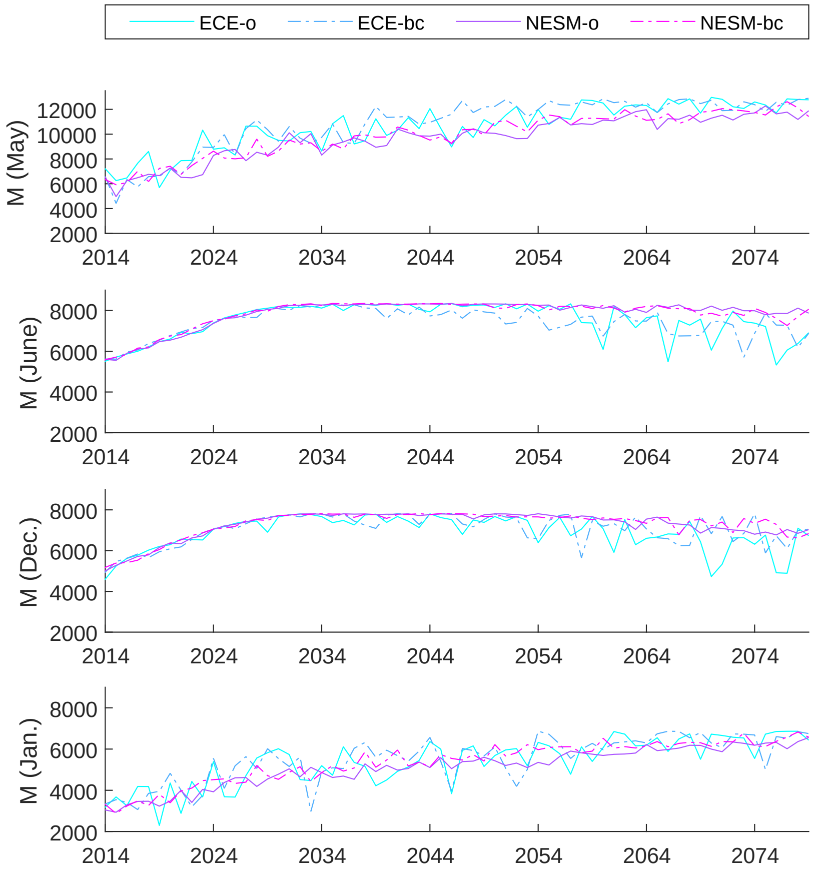

Fig. 11: Harvest pressure over time for different climate models

J Rents in the fishery

$$FS_{i\,t} = \frac{e}{1+e} c_i \left(1 + \tau_c \Delta\, t\right) \left(\frac{h_{oa\,i\,t}}{q_i(m, W_{it})\,(1-\varphi)^{1-\varphi}\, x_y^{\chi}\,(1+\tau_q \Delta\, t)} \Phi_{it}^{1-\varphi} \right)^{e+1} \tag{33}$$

$$CS_{i\,t} = \frac{\nu(B_{i\,y})}{1-\nu(B_{i\,y})} \bar{p}_i\, h_{oa\,i\,t}^{1-\nu(B_{i\,y})} \tag{34}$$

K Undiscounted rents over time

The following Table 9 shows undiscounted rents for exemplary decades associated with the four climate projections, for the BAU and melt-down policy scenarios.

Table 9: Development of undiscounted rents (in billion 2014-level-FCFA)

Model	2014–2023		2034–2043		2054–2063	
	BAU	melt-down	BAU	melt-down	BAU	melt-down
ECE-o	192	188	292	242	288	286
ECE-bc	182	179	113	131	0	0
NESM-o	191	187	147	285	0	305
NESM-bc	192	189	5	92	0	0

Additional References

99. Pech N, Samba A, Drapeau L, Sabatier R, Laloë F. Fitting a model of flexible multifleet - multispecies fisheries to Senegalese artisanal fishery data. Aquatic Living Resources. 2001;14(2):81 - 98.

100. Mbaye ML, Haensler A, Hagemann S, Gaye AT, Moseley C, Afouda A. Impact of statistical bias correction on the projected climate change signals of the regional climate model REMO over the Senegal River Basin. International Journal of Climatology. 2015;36(4):2035 - 2049. doi:10.1002/joc.4478.

101. Clark CW. Mathematical Bioeconomics. 2nd ed. New York: Wiley; 1990.
